# Chirality: a key parameter in chemical probes†

**DOI:** 10.1039/d3cb00082f

**Published:** 2023-08-08

**Authors:** Andrew McGown, Jordan Nafie, Mohammed Otayfah, Storm Hassell-Hart, Graham J. Tizzard, Simon J. Coles, Rebecca Banks, Graham P. Marsh, Hannah J. Maple, George E. Kostakis, Ilaria Proietti Silvestri, Paul Colbon, John Spencer

**Affiliations:** a Department of Chemistry, School of Life Sciences, University of Sussex Falmer BN1 9QJ UK g.kostakis@sussex.ac.uk j.spencer@sussex.ac.uk; b Sussex Drug Discovery Centre, Department of Chemistry, School of Life Sciences, University of Sussex Falmer BN1 9QJ UK; c Biotools, Inc., 17546 Beeline Highway Jupiter Florida 33458 USA; d National Crystallography Service, School of Chemistry, University of Southampton Southampton SO17 1BJ UK; e Bio-Techne (Tocris), The Watkins Building, Atlantic Road Avonmouth Bristol BS11 9QD UK hannah.maple@bio-techne.com; f Liverpool ChiroChem Ltd, The Heath Business & Technical Park Runcorn Cheshire WA7 4QX UK

## Abstract

Many small molecule bioactive and marketed drugs are chiral. They are often synthesised from commercially available chiral building blocks. However, chirality is sometimes incorrectly assigned by manufacturers with consequences for the end user ranging from: experimental irreproducibility, wasted time on synthesising the wrong product and reanalysis, to the added cost of purchasing the precursor and resynthesis of the correct stereoisomer. Further on, this could lead to loss of reputation, loss of funding, to safety and ethical concerns due to potential *in vivo* administration of the wrong form of a drug. It is our firm belief that more stringent control of chirality be provided by the supplier and, if needed, requested by the end user, to minimise the potential issues mentioned above. Certification of chirality would bring much needed confidence in chemical structure assignment and could be provided by a variety of techniques, from polarimetry, chiral HPLC, using known chiral standards, vibrational circular dichroism, and x-ray crystallography. A few case studies of our brushes with wrong chirality assignment are shown as well as some examples of what we believe to be good practice.

## Abstract 2

Chiral compounds are vital as chemical probes. Choosing a wrong enantiomer (mirror image) can be disastrous in a medicinal chemistry programme. Here, a male kingfisher emerges from the water with its “active form” above the surface.

## Graphical abstract

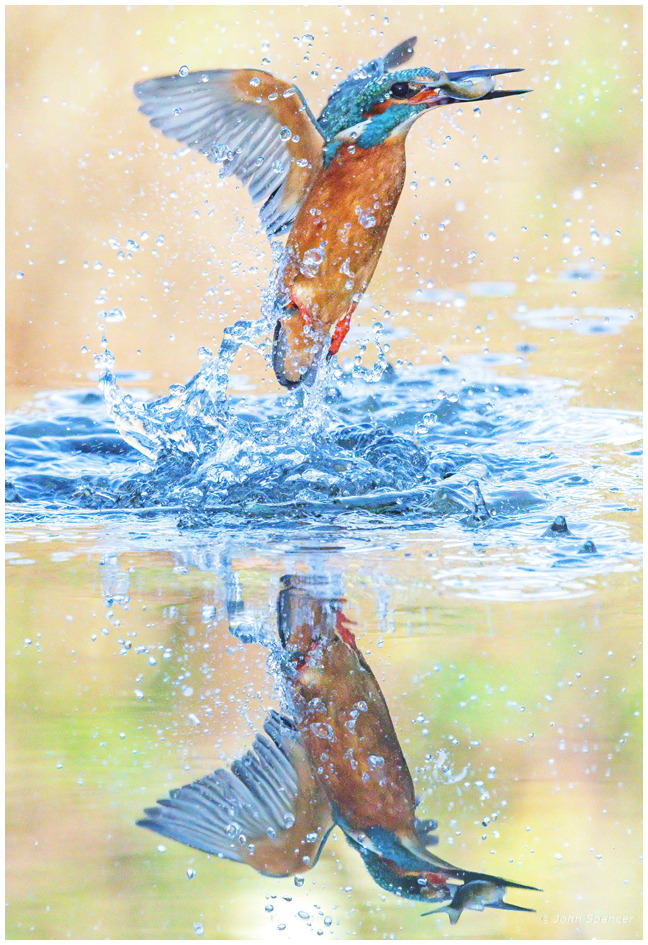

## Introduction

**Fig. 1:**
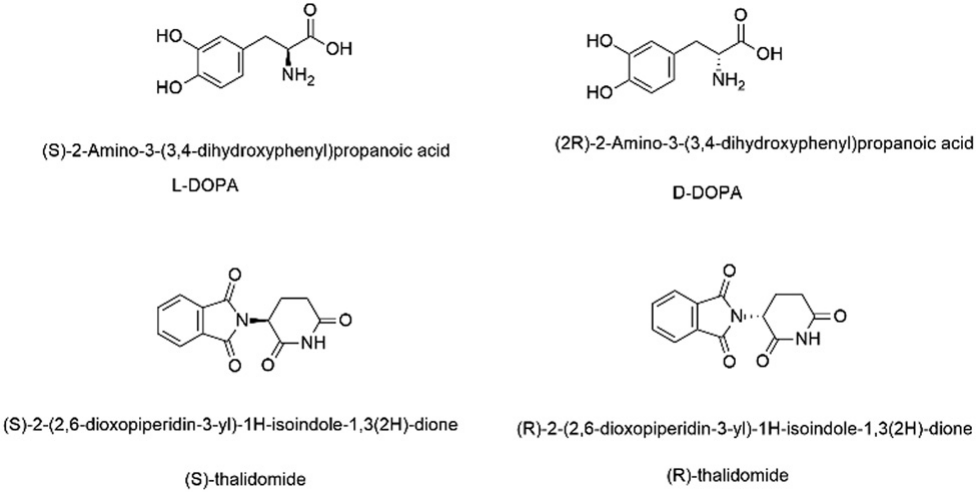
Examples of chiral drugs and their enantiomers.

Chemical probes (or “tool compounds”) can be defined as small molecules with known pharmacological activity, that selectively and potently modulate the activity of a target protein. As such, they provide invaluable mechanistic and phenotypic insights into the proteome that can validate new drug targets and progress our understanding of disease-relevant pathways.^1^ Published research from studies that employ chemical probes contributes to our collective scientific understanding, helping to shape the direction of research programs and entire fields. A framework of “fitness factor” guidelines for chemical probes has therefore been proposed to ensure the validity and reproducibility of such research.^2^ These ‘fitness factors’ include, *inter alia*: criteria around potency and selectivity; the observation of structure activity relationships (SAR) across a series, *i.e.* not a “singleton” hit or a pan-assay interference (PAIN) compound;^3^ identification of a chemotype-matched negative control; known chemical stability, solubility and permeability; and absolute definition of a discrete chemical structure, including stereochemistry. Different stereoisomers of the same compound can profoundly alter potency, selectivity, mechanism of action as well as pharmacokinetic parameters such as half-life, clearance, and toxicity.^4,5^ A clear example of the importance of chirality in chemical probes is the observation that chemotype-matched negative control compounds are very often an inactive enantiomer of the chiral probe molecule.^6^ Chirality is increasingly vital for 3D chemical space exploitation in drug design and key to biological activity.^7^ Hence, it is vital to ensure that a chiral molecule's absolute stereochemistry is correctly assigned and indeed this is a basic premise in synthetic organic chemistry. As early as undergraduate level, chemists are taught about stereochemistry and its consequences in a medicinal context:^8^l-, not d-, Dopa is an anti-Parkinson's drug,^9^ and thalidomide was administered as a morning sickness drug in racemic form, with terrible consequences (Fig. 1).^10^ Thalidomide analogues have undergone a dramatic revival as anticancer drugs, but racemisation often occurs in biological matrices.^11^

We present a few recent personal “horror shows”, where chiral chemical probes purchased from a commercial provider were incorrectly assigned. We strongly recommend that the responsibility lies with the manufacturer to ensure that stereochemical assignment is correct and is certified using analytical chemical methods such as: vibrational circular dichroism (VCD);^12–14^ polarimetry, the use of a shift reagent;^15^ chiral HPLC (with a known standard *e.g.* using the opposite enantiomer as a control to show analytical separation of the enantiomers);^16^ or X-ray crystallography.^17^ We present this case study from an academic, or *e.g.* spin-out/start-up industrial viewpoint, where resources (*e.g.* researcher time, in house facilities and technical support) are likely to be much more limited than in a larger research organisation. Larger pharmaceutical organisations, routinely conduct their own due diligence on any chemical (or biological) entities) and are highly “immune” to the issues pointed out below.

## Case study #1. (+)-JQ1 or not (+)-JQ1?

**Fig. 2:**
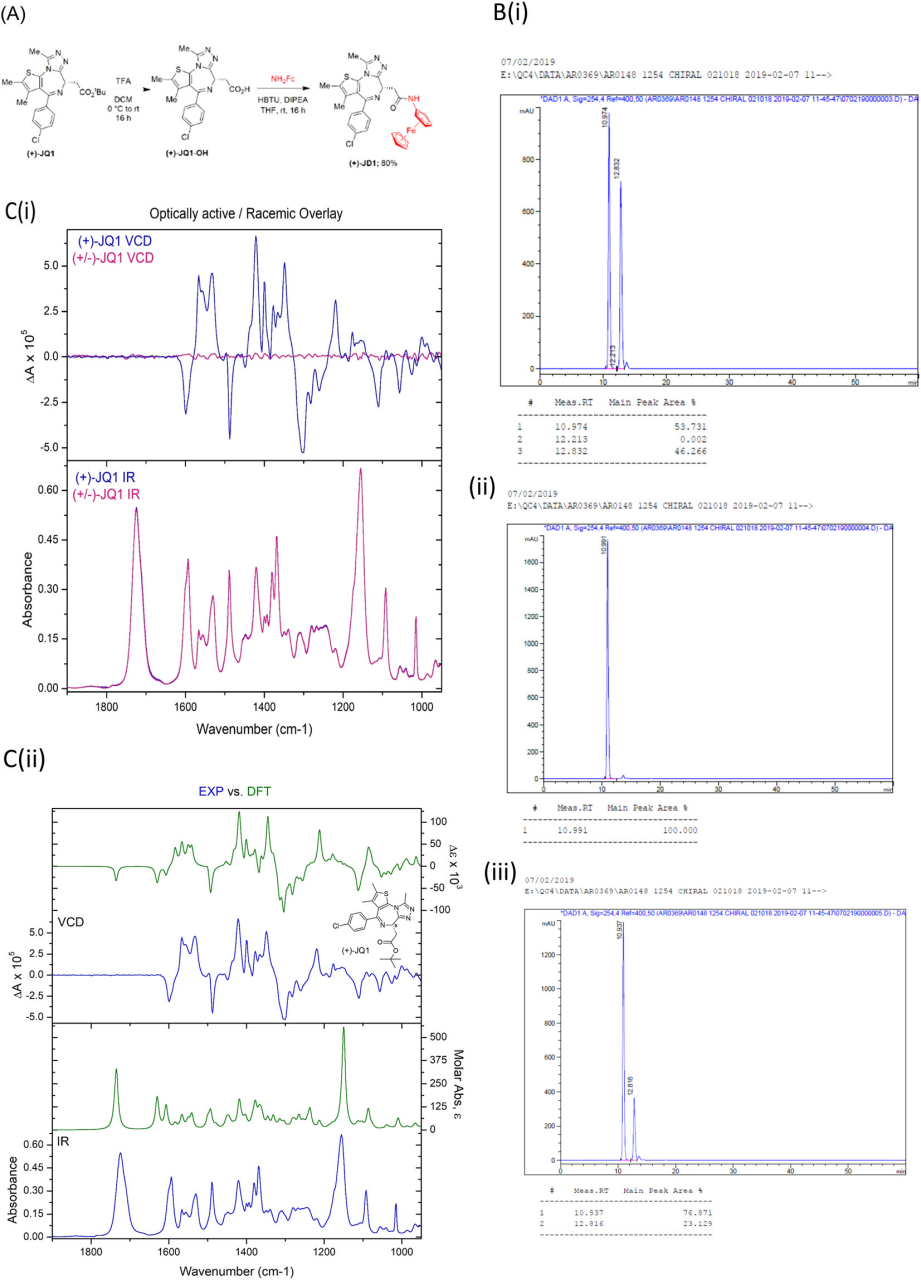
(A) Synthesis of (+)-JD1. (B) HPLC chromatograms of the initially procured commercial “(+)-JQ1” ((i) *R*_t_ = 10.9 and (ii) 12.9 min), (iii) authentic (+)-JQ1 from Bio-Techne (Tocris) (*R*_t_ = 10.9 min), and a co-injection of both samples (right). (C) (i) VCD of (+)-JQ1 *vs*. rac.JQ1 (ii) (+)-JQ1 experimental VCD and calculated.

The BRD BET bromodomain inhibitor (+)-JQ1 is an established tool molecule in epigenetics. It is a chiral molecule, whose opposite enantiomer, (−)-JQ1, is inactive and serves as a negative control.^6^ Recently, we synthesised an organometallic (aminoferrocene (NH_2_Fc)) analogue of (+)-JQ1 termed (+)-JD1 (Fig. 2A).^18^ Starting from a commercial source of “(+)-JQ1”, ester deprotection followed by a simple amide coupling afforded the desired compound in good yield. Analysis of the product by chiral HPLC, however, revealed that the product of this reaction was in fact a 1 : 1 mixture of enantiomers. Subsequent analysis of the commercial molecule advertised as (+)-JQ1, by chiral HPLC, revealed that it was in fact a racemic mixture Fig. 2B(i)). This was a surprising finding since the supplier in question had provided a ‘single peak’ chiral HPLC analysis. The entire synthesis had to be repeated using a genuine commercial sample of (+)-JQ1 obtained from Bio-Techne (Tocris). We validated the absolute chirality of this new commercial source of (+)-JQ1 by chiral HPLC (Fig. 2B(ii) and (iii)) and VCD (Fig. 2C), before resynthesizing (+)-JD1. The supply of racemic JQ1, mis-advertised as (+)-JQ1 from our original vendor, wasted time and resources, and it is likely that other research groups have been unknowingly using racemic JQ1 in their studies and publishing misleading results.

In this example, we were able to use chiral HPLC to provide absolute stereochemical determination of structure because we had access to a reference sample of known chirality, as well as its enantiomer with different *R*_t_ values, as controls. However, absolute configuration is not always known, and controls are not always available. In these instances, VCD or measurement of optical rotation by polarimetry, can be useful to ensure that the correct single enantiomer has been obtained. The sample of (+)-JQ1 from Bio-Techne (Tocris) was provided with a certificate of analysis which clearly stated the purity by both HPLC and chiral HPLC, and provided optical rotation data for the compound to prove identity and chiral purity. It is important for researchers to have access to or expertise in, the analytical methods used here to check the purity and identity of purchased chemical probes. We would urge researchers to check that this information has been provided with commercial compounds and exercise caution in their choice of provider.

## Case study #2. Is it (*R*)-,(*R*)- or (*S*)-,(*S*)-?

**Fig. 3:**
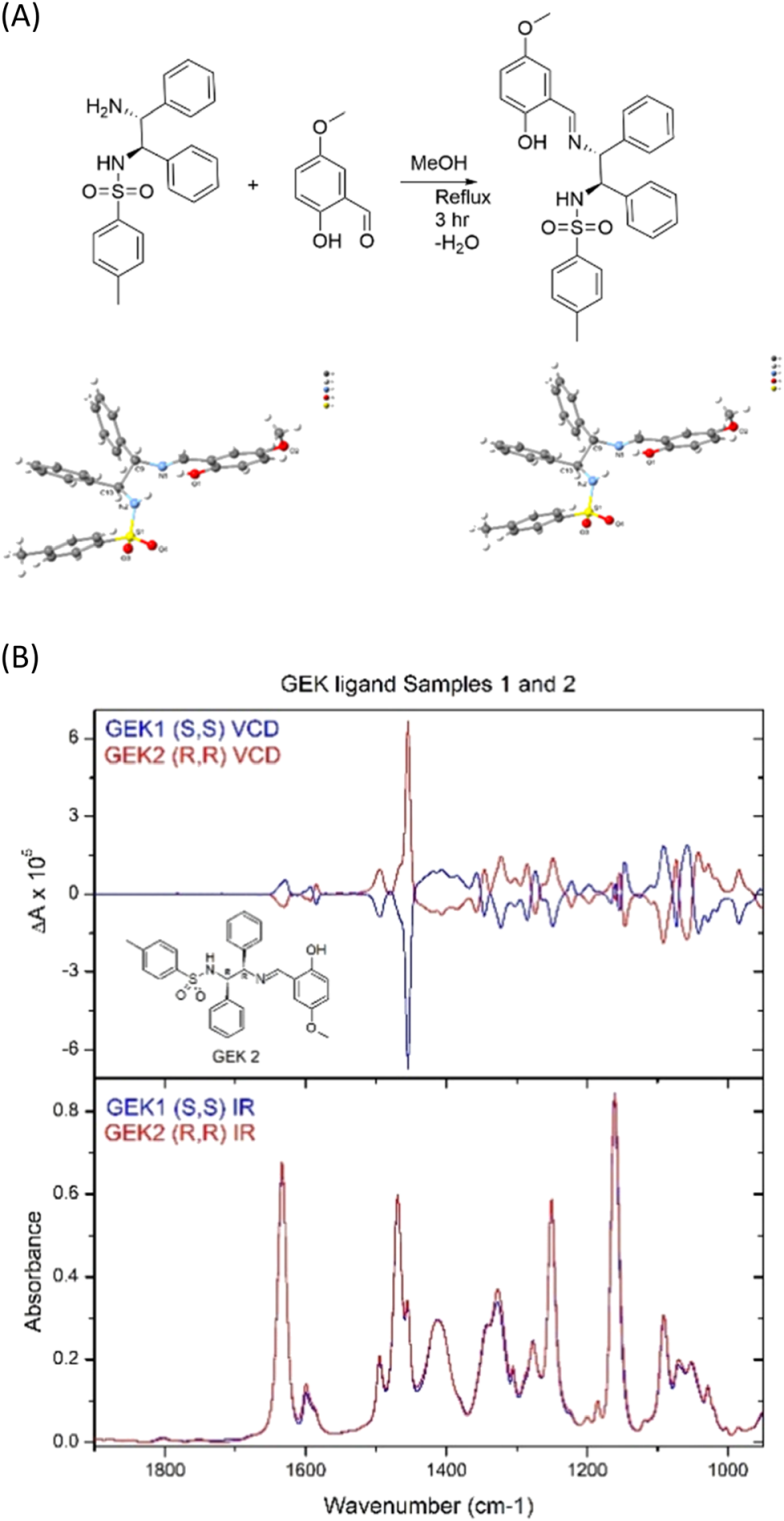
(A) Synthesis of 1(*S*),2(*S*)-*N*,*N* chiral ligand for catalysis and projection of the crystal structure of the chiral (*S*)-,(*S*)- (left) and (*R*)-,(*R*)- (right) organic ligands. (B) VCD spectra of (*R*)-,(*R*)- and (*S*)-,(*S*)- ligands.

Converting a *trans*-1,2-diaminocyclohexane ligand of known chirality, at carbon, to form a metal complex for catalysis *via N*,*N*-coordination is usually straightforward.^19^ However, when the absolute stereochemistry of the carbon backbone of the ligand, not involved in any reaction, has seemingly undergone a double inversion, upon metal complexation, this raises many questions, *e.g.*, did the student use the right starting material? This can cause loss of confidence, breakdown in group dynamics, loss of time, unnecessary stress brought upon a relatively inexperienced researcher. In our case, several X-ray attempts showed simply that our commercial (*R*)-,(*R*)-sample was incorrectly labelled as (*S*)-,(*S*)-. X-ray crystallography, then VCD, enabled us to assign unambiguous stereochemistry to both enantiomers (Fig. 3).

## Case study #3. The way forward?

**Fig. 4:**
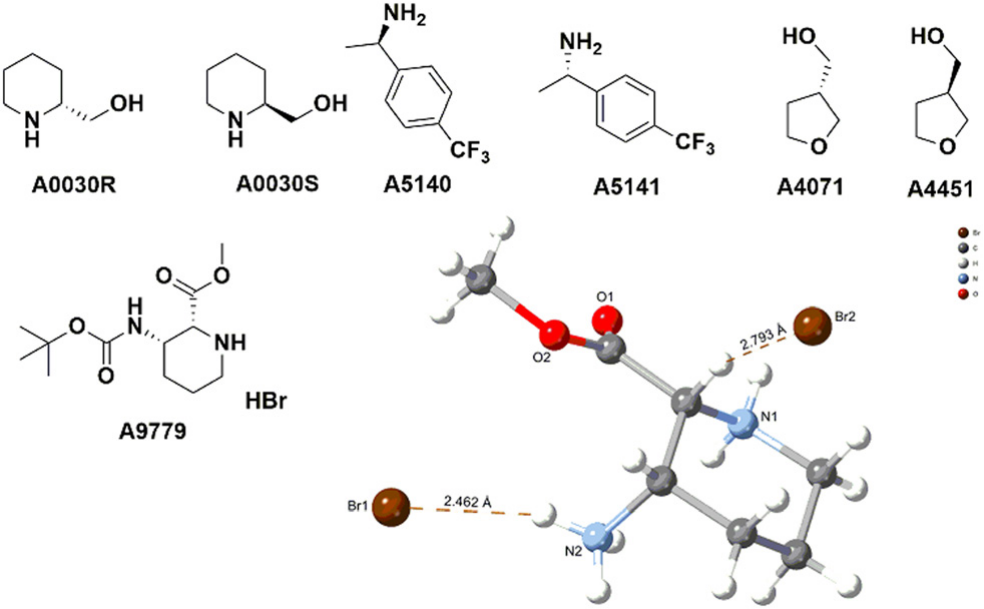
Selected examples of LCC stock compounds with known absolute configuration.

The absolute configuration of chiral compounds at Liverpool Chirochem (LCC) was previously determined by X-ray or by comparison with data reported in the literature. Often, it was necessary to recrystallise the compounds of interest multiple times and/or derivatise them to obtain crystals of sufficient quality for the analysis. When the company first introduced the use of VCD for the determination of absolute configuration, a set of enantiopairs with known configuration was tested. Results were all in agreement with the previous assignment with high confidence score. Only compounds in stock as free bases (*i.e.*, no HCl/TFA salts) were included in the set and carboxylic acids were not tested (Fig. 4). Compounds are provided with a certificate of analysis.

**Fig. 5:**
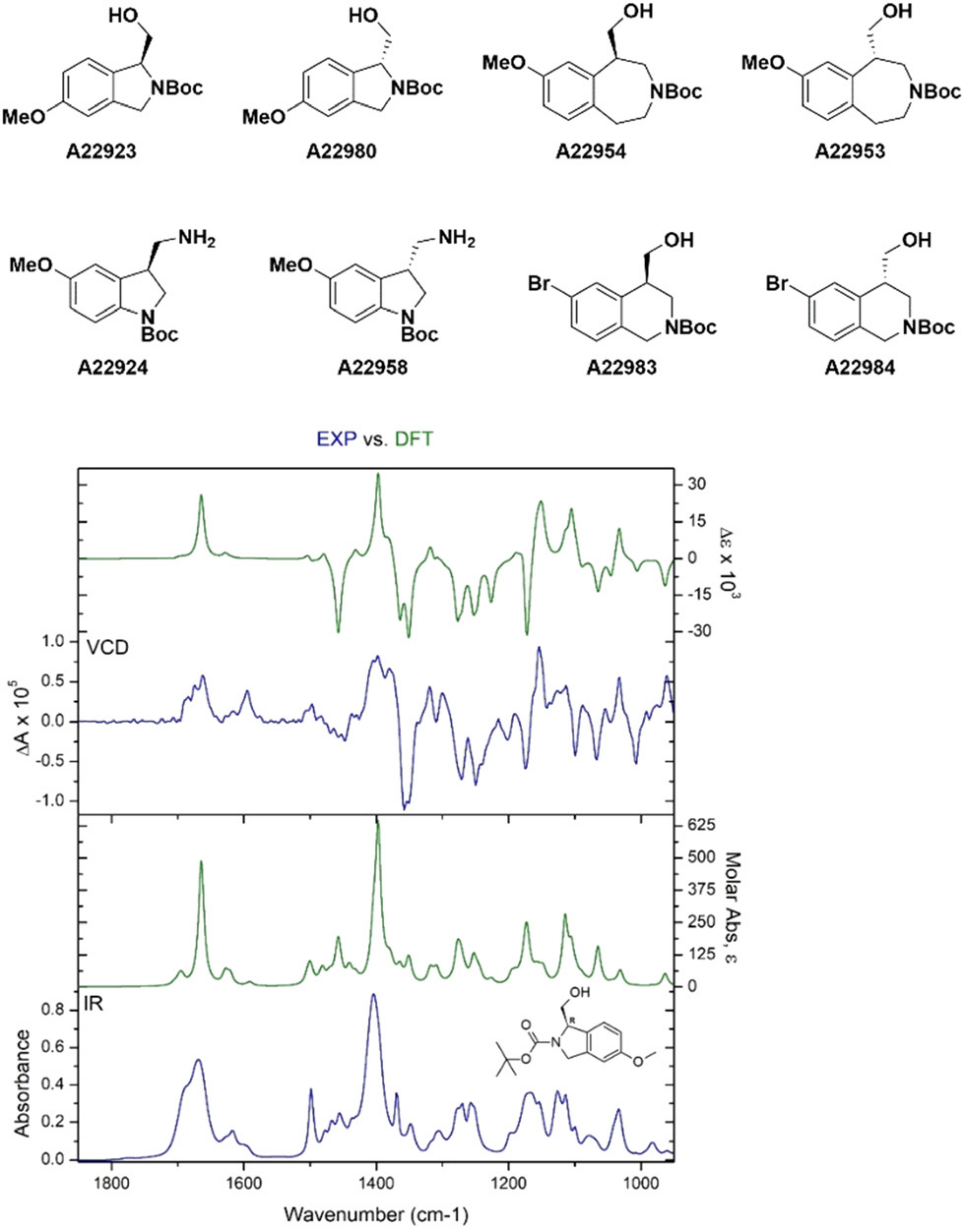
Selected examples of novel building blocks requiring determination of absolute configuration. Bottom figure shows A2292*3* structural assignment by VCD.

Recently, a series of novel, multifunctionalised, chiral building blocks were designed and synthesised and required the determination of absolute configuration. As all compounds were oils, VCD was the technique of choice (Fig. 5).

Adopting VCD as a technique has allowed LCC to confidently assign absolute configuration with no sample manipulation. Oils and amorphous solids could be analysed as well without the need of derivatisation or lengthy crystallisations. For compounds with an average molecular weight between 250–350 Da, the analysis requires about 10 mg of materials, which could be recovered if necessary.

## Case study #4. It is not just about chirality!

**Scheme 1:**
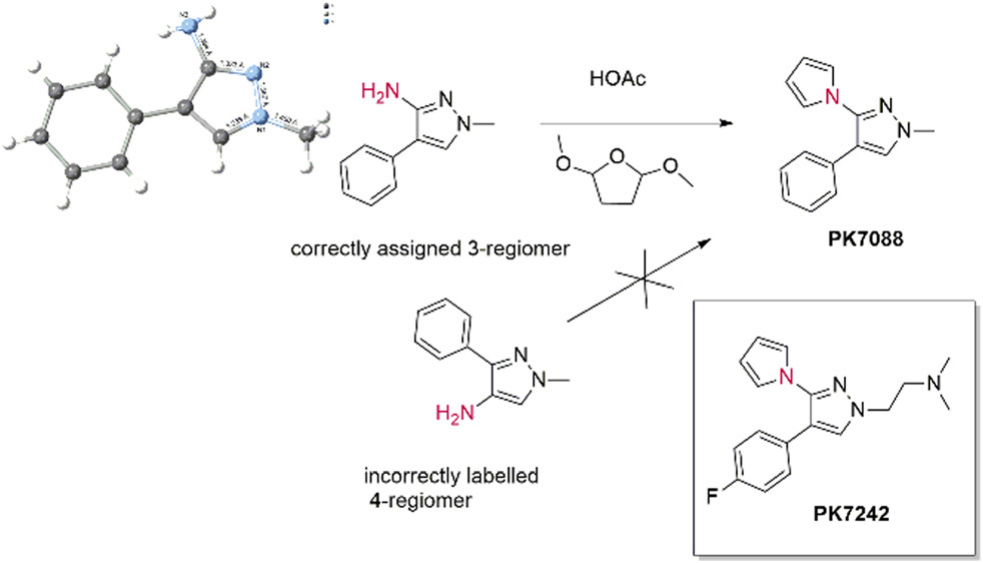
A 3-aminopyrazole yields a 3-aminopyrazole product, not the 4-regioisomer, as expected from the label.

We have much trust in what we buy and what is on the label, in very much the same way as believing that a colourless liquid in a sealed water bottle labelled “water” is indeed water and not some noxious material. In chemistry terms, despite having an aldehyde on the label, the contents might be its unoxidized alcohol precursor plus unreacted, malodorous Swern reagents (we have experienced this recently), or the isomer on the label might not be the actual product. As a final case study, we cite a non-chiral example where a chemical probe was incorrectly labelled in terms of regiochemistry. PK7088 (1-methyl-4-phenyl-3-(*1H*-pyrrol-1-yl)-1*H*-pyrazole) is an stabiliser of mutant p53-Y220C (large to small; tyrosine to cysteine).^20^ We synthesised PK7088 in house and found that the regiochemistry of the product appeared in conflict with the “4-amino-pyrazole” precursor that we had purchased. An x-ray co-crystal structure (pdb: 3ZME) of PK7242, made from a similar precursor to PK7088, bound to a Y220C-p53, established that the correct regiochemistry was that of a 3-aminopyrazole. Consequent docking showed that only this 3-isomer was able to bind to the protein pocket. To finally solve this conundrum, directly, we were able to crystallise the commercial precursor to PK7088 to establish the correct regiochemistry, showing it to be the 3-regioisomer, 1-methyl-4-phenyl-3-aminopyrazole, not the incorrectly labelled 4-regioisomer (Scheme 1).^21^ However, we never recovered the hours of lost time trying to solve this conundrum.

## Conclusions

The importance of correctly assigned chirality (and other forms of isomerism) are undisputed in the pharmaceutical industry. The well-known tragic example of thalidomide as a drug for morning sickness illustrates the potential consequences of inattention to this detail, ultimately resulting from differential biological activity from different isomers. It follows that chemical probe developers and manufacturers should pay close attention to chiral identity and purity. We have, however, experienced several instances where such attention has not been paid, resulting in lost time and wasted resource. We further postulate that we are not alone in these experiences, and that other research groups may be unknowingly using structurally incorrect or impure chemical probes in their work. This is highly damaging for the validity and reproducibility of the resulting scientific literature, and may lead to paper retractions, corrections, or IP (intellectual property) loss. The importance of chirality extends to chemical building blocks, which may be further elaborated to a final drug lead or candidate with the wrong stereochemistry, over several expensive steps. The mistake may be revealed by the absence or loss, of the expected biological activity, or indeed toxicity, following expensive assays, where animals may be involved. This could result in potential ethical and safety concerns, loss of funding (the research fellow's contract might have ended before the resynthesis is carried out), lengthy corrective action through expensive resynthesis and stereochemistry reassignment, loss of reputation, and unnecessary animal experiments. We advocate for continued awareness of this issue and strongly recommend that commercial suppliers (and not the end user) perform stringent quality control and be able to certify chiral purity. We recommend that researchers perform their own due diligence on suppliers, and purchase from reputable companies who conform to high standards of quality and integrity. Whereas large pharmaceutical companies adhering to good manufacturing practice (GMP) will avoid many of these issues, academic groups will need to demand full analyses, build trust in good suppliers, avoid bad ones, to ensure that their probes are genuine and correctly assigned. Our recommendations extend to, building in-house, a database of preferred suppliers (this might change over time if suppliers drop their standards), adding them to the procurement/preferred suppliers list on the financial management system, establishing a rapport between such vendors, *e.g.* a local representative, so that any technical troubleshooting may be resolved by a quick phone call or email conversation. This builds up more trust between vendor and end user; indeed, they might make you custom probes on demand, that are otherwise not in their catalogue. On the flipside, we need to feedback to suppliers who continue to provide substandard products that “enough is enough” and stop using them unless they improve their standards. Practically, we recommend use of VCD, polarimetry, x-ray crystallography or chiral HPLC (of the desired compound together with opposite enantiomer or racemic mixture; a single peak on chiral HPLC may not signify a single enantiomer!) to characterize chiral molecules. This work is hopefully a useful addition to other similar recent publications advocating good practice in chemical analysis and compound purity.^22,23^

## Author contributions

JS conceived this study and wrote the paper with major input from GEK, HJM and all others’ comments and final consent to submit. JN carried out VCD measurements and calculations. AM, MO, SHH carried out synthetic chemistry and GJT, GEK and SJC performed Xray structural determination and analysis. GPM, HJM and RB performed chiral HPLC analysis and data interpretation. IPS and PC carried out analysis of the LCC collection.

## Conflicts of interest

There are no conflicts to declare.

## Supplementary Material

CB-004-D3CB00082F-s001

CB-004-D3CB00082F-s002
