# Supplementary material for: Chirality: a key parameter in chemical probes
Source: RSC Chem Biol. 2023 Aug 8;4(10):716–21. doi: 10.1039/d3cb00082f (PMC10549247; doi:10.1039/d3cb00082f)
Supplement: CB-004-D3CB00082F-s001 [file CB-004-D3CB00082F-s001.pdf]

Electronic Supplementary Material (ESI) for RSC Chemical Biology.
This journal is © The Royal Society of Chemistry 2023

Chirality: a Key Parameter in Chemical Probes

Andrew McGown,[a,b] Jordan Nafie,[c] Mohammed Otayfah,[a] Storm Hassell-Hart,[a] Graham J. Tizzard,[d] Simon J. Coles,[d] Rebecca Banks,[e] Graham P. Marsh,[e] Hannah J. Maple*,[e] George E. Kostakis*,[a] Ilaria Proietti Silvestri,[f] Paul Colbon,[f] John Spencer*,[a,b]

[a] Department of Chemistry, School of Life Sciences, University of Sussex, Falmer, BN1 9QJ, UK.
[b] Sussex Drug Discovery Centre, Department of Chemistry, School of Life Sciences, University of Sussex, Falmer, BN1 9QJ, UK.
[c] Biotools, Inc., 17546 Beeline Highway, Jupiter, Florida 33458, US.
[d] National Crystallography Service, School of Chemistry, University of Southampton, Southampton, SO17 1BJ, UK.
[e] Bio-Techne (Tocris), The Watkins Building, Atlantic Road, Avonmouth, Bristol BS11 9QD, UK.
[f] Liverpool ChiroChem Ltd, The Heath Business & Technical Park, Runcorn, Cheshire WA7 4QX, UK.

Contents

Xray structures and table of data…………………………………………………… Pages S1-S4
Synthesis and Characterisation of Ligands……………………………………………. S5-S7
VCD details……………………………………………………………………………………….. S8-S42
References……………………………………………………………………………………….. S42

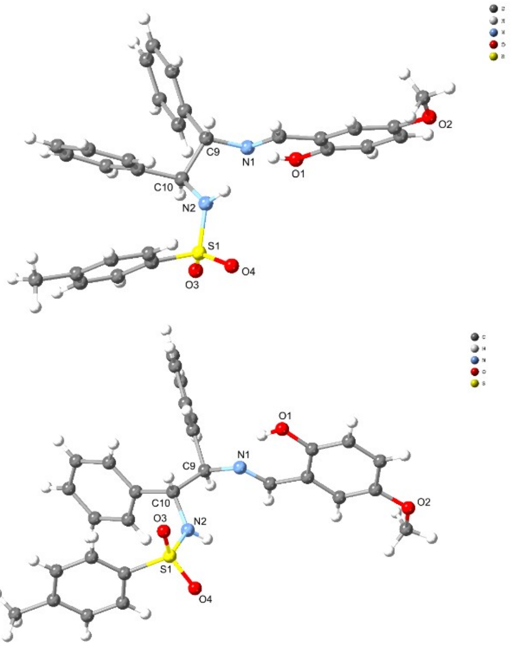

Figure S1. A projection of the crystal structure of the chiral SS (upper) and RR (lower) organic ligands.

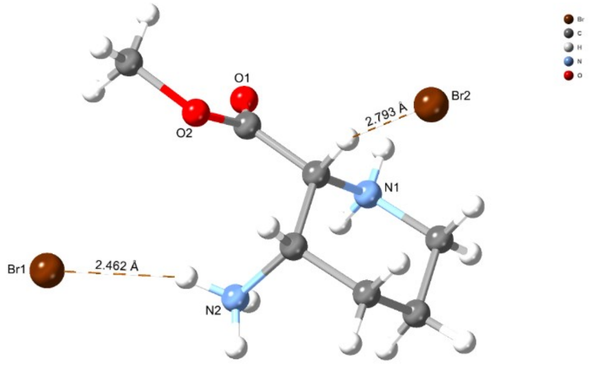

Figure S2: A9779 structure

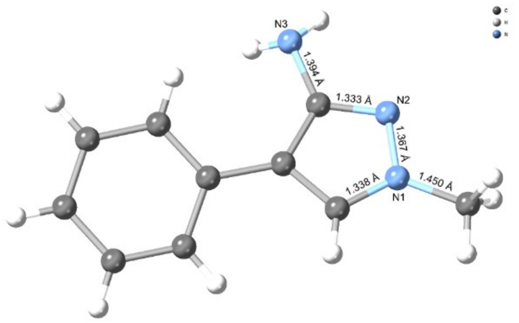

Figure S3: 3-aminopyrazole regiomer by x-ray

Table S1 Crystal data and structure refinement for the 4 compounds

Identification code	**SS**	**RR**	**3-Aminopyrazole analogue**	LCC- A9779
Empirical formula	$C_{29}H_{28}N_2O_4S$	$C_{29}H_{28}N_2O_4S$	$C_{10}H_{11}N_3$	$C_7H_{16}Br_2N_2O_2$
Formula weight	500.59	500.59	173.219	320.04
Temperature/K	100.0(6)	100.01(10)	100(2)	150.0
Crystal system	orthorhombic	orthorhombic	orthorhombic	orthorhombic
Space group	$P2_12_12_1$	$P2_12_12_1$	$P2_12_12_1$	$P2_12_12_1$
a/Å	6.44134(7)	6.43966(10)	5.9358(2)	9.1475(6)
b/Å	17.00295(19)	16.9994(3)	8.3325(3)	10.8895(8)
c/Å	23.3012(2)	23.3008(7)	17.6337(6)	12.2544(9)
α/°	90	90	90	90
β/°	90	90	90	90
γ/°	90	90	90	90
Volume/Å^3	2551.99(5)	2550.74(10)	872.16(5)	1220.68(15)
Z	4	4	4	4
ρ_{calc}g/cm^3	1.303	1.304	1.319	1.741
μ/mm^{-1}	1.435	1.436	0.083	8.256
F(000)	1056.0	1056.0	368.2	632.0
Crystal size/mm^3	0.12 × 0.1 × 0.05	0.1 × 0.08 × 0.03	0.09 × 0.09 × 0.03	0.22 × 0.18 × 0.08
Radiation	Cu Kα (λ = 1.54184)	Cu Kα (λ = 1.54184)	Mo Kα (λ = 0.71075)	CuKα (λ = 1.54178)
2Θ range for data collection/°	7.588 to 142.826	10.408 to 134.17	5.4 to 55.2	10.868 to 140.058
Index ranges	-7 ≤ h ≤ 7, -20 ≤ k ≤ 20, -28 ≤ l ≤ 24	-7 ≤ h ≤ 7, -19 ≤ k ≤ 20, -23 ≤ l ≤ 27	-7 ≤ h ≤ 7, -10 ≤ k ≤ 10, -22 ≤ l ≤ 22	-11 ≤ h ≤ 11, -12 ≤ k ≤ 13, -13 ≤ l ≤ 14
Reflections collected	13429	14476	11078	5642
Independent reflections	4668 [R_{int} = 0.0340, R_{sigma} = 0.0300]	4451 [R_{int} = 0.0288, R_{sigma} = 0.0210]	2018 [R_{int} = 0.0364, R_{sigma} = 0.0222]	2220 [R_{int} = 0.0439, R_{sigma} = 0.0489]
Data/restraints/parameters	4668/767/328	4451/0/328	2018/0/120	2220/0/121
Goodness-of-fit on F^2	1.134	1.055	1.065	1.140
Final R indexes [I>=2σ (I)]	R_1 = 0.0311, wR_2 = 0.0819	R_1 = 0.0274, wR_2 = 0.0714	R_1 = 0.0325, wR_2 = 0.0798	R_1 = 0.0394, wR_2 = 0.1170
Final R indexes [all data]	R_1 = 0.0329, wR_2 = 0.0834	R_1 = 0.0274, wR_2 = 0.0714	R_1 = 0.0349, wR_2 = 0.0810	R_1 = 0.0395, wR_2 = 0.1172
Largest diff. peak/hole / e Å^{-3}	0.32/-0.30	0.26/-0.26	0.18/-0.18	0.80/-0.82
Flack parameter	-0.004(7)	-0.007(6)	0.0(9)	-0.01(3)

Experimental section

Materials.

All reagents were purchased from Fluorochem, Fischer Scientific or Alfa Aesar and used without further purification. Experiments were performed under aerobic conditions.

Instrumentation

NMR spectra were recorded with a Varian VNMRS 600 at 25 °C, at either 600 MHz or 151 MHz in Chloroform-d or DMSO-d_6. Chemical shifts are quoted in parts per million (ppm). Coupling constants (J) are recorded in units of Hz. FT-IR spectra were recorded over the range of 4000–650 cm^{-1} on a PerkinElmer Spectrum One FT-IR spectrometer fitted with a UATR polarisation accessory. HRMS data were obtained with a Bruker Daltonics Fourier Transform (FTMS) Apex II spectrometer with electrospray ionization (ESI) and methanol as solvent. HR-MS data were obtained on a VG Autospec Fissions instrument (EI at 70 eV). Molecular ions are reported as mass/charge (m/z) ratios.

Single crystal X-Ray Diffraction

Crystallographic data for **SS** and **RR** were collected (ω scans) at the University of Sussex using an Agilent Xcalibur Eos Gemini Ultra diffractometer with CCD plate detector under a flow of nitrogen gas at 100(2) K and Cu Kα radiation (λ =1.54184 Å). The data were processed with CrysAlisPro and solved by intrinsic phasing methods with SHELXT.[1] All crystal structures were then refined on Fo^2 by full-matrix least-squares refinements using SHELXL.[1] Geometric/crystallographic calculations were performed using PLATON,[2] Olex2,[3] and WINGX[4] packages; graphics were prepared with CrystalMaker.[5]

Ligand synthesis

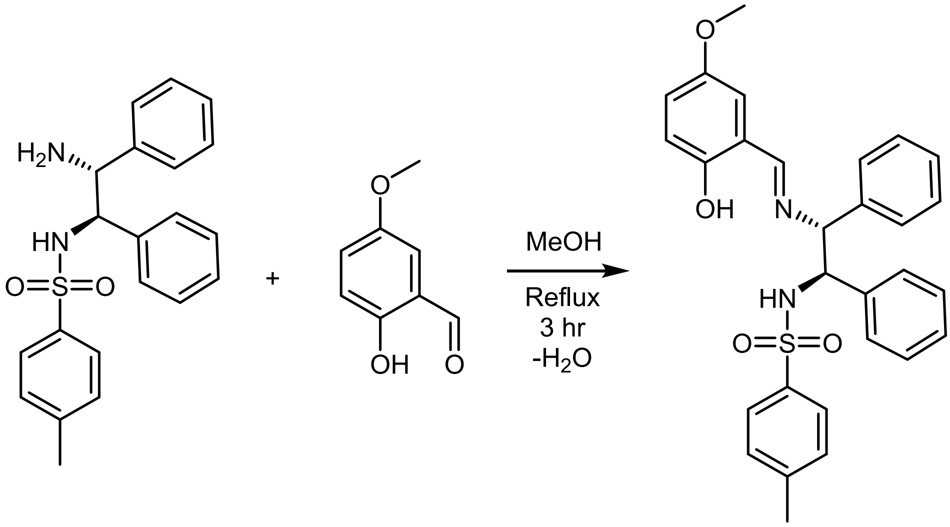

Scheme S1. Synthesis of N-[(1S,2S)-2-[(E)-[(2-hydroxy-5-methoxyphenyl)methylidene]amino]-1,2-diphenylethyl}-4-methylbenzene-1-sulfonamide. (SS)

(1S,2S) -(-)-N-(4-Toluenesulfonyl)-1,2-diphenylethylenediamine (0.4465 g, 1.2mmol, 1 eq) and 2-Hydroxy-5-methoxybenzaldehyde (0.15 ml, 1.2mmol, 1 eq) were dissolved in 20ml of methanol and heated (80°C) in a round bottom flask for 3hr.The the reaction was cooled to RT and the excess solvent was removed in vacuo. The crude material was washed with cold EtOH (ca. 100 mL) and dried to afford the SS ligand. **ESI-ETMS** in methanol: m/z cald. for [$C_{29}H_{30}N_2O_4S$] : 502.63 found 502.07 . Yield =1.4166 g (78%). **^{1}H NMR** [1] (600 MHz, Chloroform-*d*) δ 12.18 (s, 1H), 8.12 (s, 1H), 7.42 – 7.37 (m, 2H), 7.20 (dd, *J* = 5.1, 2.0 Hz, 3H), 7.16 – 7.07 (m, 5H), 7.00 (d, *J* = 8.0 Hz, 2H), 6.97 – 6.86 (m, 4H), 6.62 (d, *J* = 3.0 Hz, 1H), 5.21 (d, *J* = 7.4 Hz, 1H), 4.76 (dd, *J* = 7.4, 5.6 Hz, 1H), 4.57 (d, *J* = 5.6 Hz, 1H), 3.73 (s, 3H), 2.31 (s, 3H). **^{13}C NMR** (151 MHz, Chloroform-*d*) δ 166.9, 154.9, 143.0, 138.2, 129.2, 128.4, 128.0, 127.9, 127.6, 127.6, 127.5, 126.9, 120.1, 118.0, 117.7, 115.1, 78.4, 63.3, 55.8, 21.4.

Synthesis of N-[(1R,2R)-2-[(E)-[(2-hydroxy-5-methoxyphenyl)methylidene]amino]-1,2-diphenylethyl}-4-methylbenzene-1-sulfonamide.(RR) (1R,2R) -(-)-N-(4-Toluenesulfonyl)-1,2-

diphenylethylenediamine (0.4465 g, 1.2mmol, 1 eq) and 2-Hydroxy-5-methoxybenzaldehyde (0.15 ml, 1.2mmol, 1 eq) were dissolved in 20ml of methanol and heated (80°C) in a round bottom flask for 3hr.The the reaction was cooled to RT and the excess solvent was removed in vacuo. The crude material was washed with cold EtOH (ca. 100 mL) and dried to afford the RR ligand. **ESI-ETMS** in methanol: m/z cald. for [$C_{29}H_{30}N_2O_4S$] : 502.63 found 502.07 . **^{1}H NMR** ^{1}H NMR (500 MHz, DMSO-d_6) δ 12.20 (s, 1H), 8.47 (d, *J* = 8.1 Hz, 1H), 8.42 (s, 1H), 7.30 (d, *J* = 7.9 Hz, 2H), 7.13 (d, *J* = 4.2 Hz, 4H), 7.09 (q, *J* = 4.4 Hz, 1H), 6.96 (dq, *J* = 22.6, 4.7, 4.2 Hz, 9H), 6.81 (d, *J* = 8.8 Hz, 1H), 4.77 (t, *J* = 7.9 Hz, 1H), 4.50 (d, *J* = 8.7 Hz, 1H), 3.70 (s, 3H), 2.17 (s, 3H).**^{13}C NMR** (151 MHz, cdcl$_3$) δ 166.9, 154.9, 152.1, 143.0, 137.3, 136.8, 129.3, 128.5, 128.0, 127.9, 127.6, 127.6, 127.5, 126.9, 120.2, 117.8, 115.1, 78.4, 77.2, 77.0, 76.8, 63.3, 55.8, 21.4.

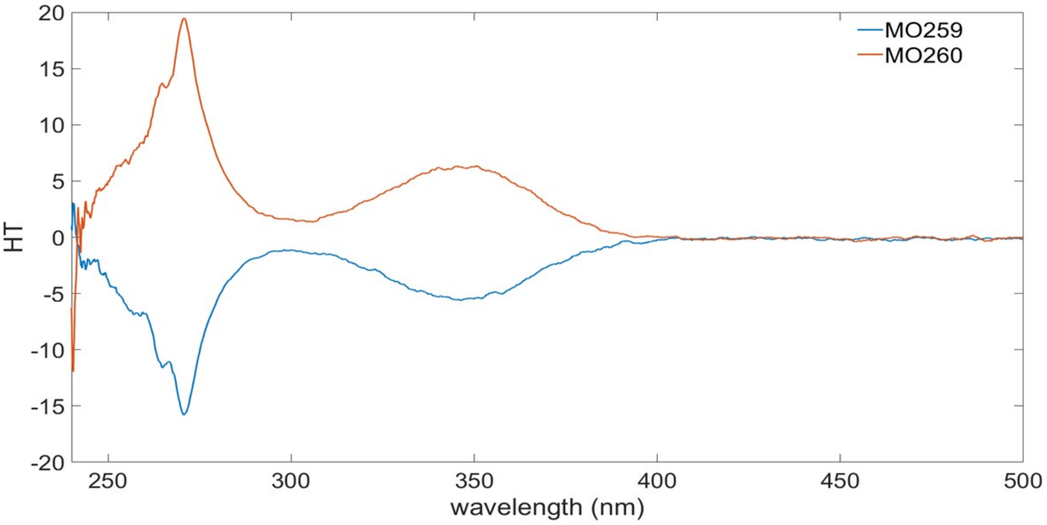

Figure

Figure S4. The CD spectra for **SS** (MO259) and **RR** (MO260) ligands recorded in MeOH 1mM.

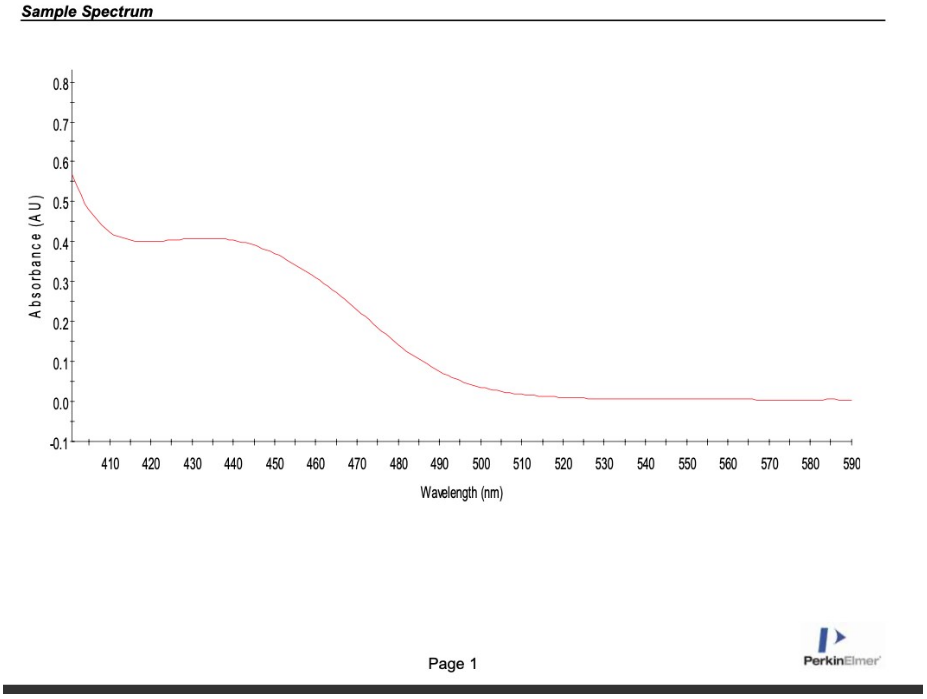

Figure S5. The UV-Vis spectra for **SS** (MO259) and **RR** (MO260) ligands recorded in MeOH 1mM.

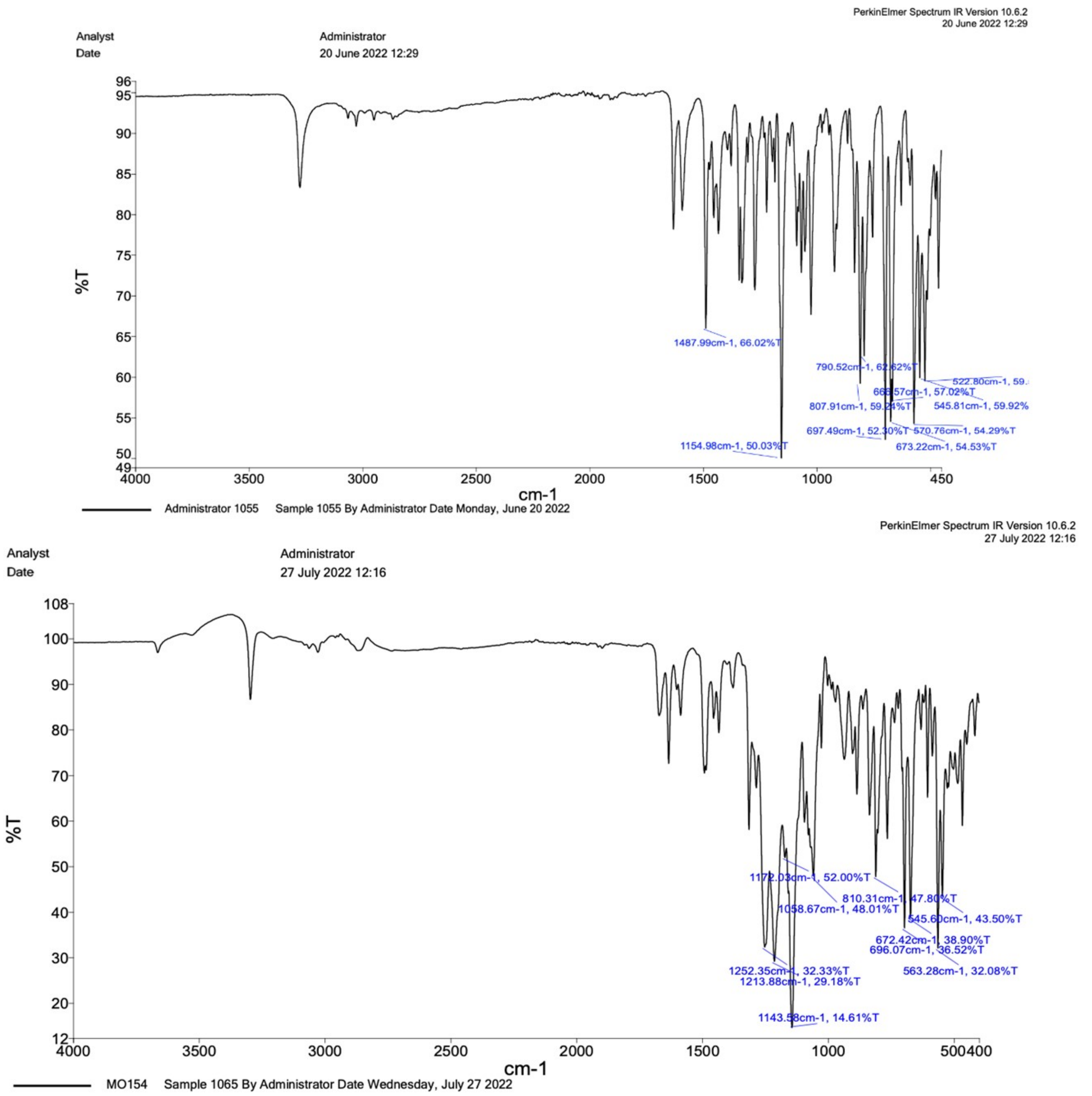

Figure S6. The IR spectra for **SS** (MO259) and **RR** (MO260) ligands recorded in solid state

General methods for absolute configuration determination by VCD.

The absolute configuration (AC) of a molecule can be determined using IR / VCD spectroscopy by comparing the experimental spectra to the *ab initio* DFT calculated IR / VCD spectra of a chosen enantiomer[6-8]. The experimental side is straightforward, the important factors being the solvent and the concentration (for a given cell path). Generally, NMR solvents are used in order to maximize the available spectral window in the infrared fingerprint region, with $CDCl_3$ being the chosen solvent as long as the chiral compound is soluble. This solvent has a broad spectral window (950 – 2000 cm^{-1}) and has minimal solvent-solute interactions, which is favorable for getting DFT calculations to match with the experiment. The concentration is determined experimentally, with the optimal range of absorbances being 0.2 – 0.8 A for the region of interest (typically the fingerprint region). Practically, the concentration is adjusted as high as possible while still keeping the most intense IR peaks below 1.0 A. Once the solution is prepared, the measurement is taken in 1 hour blocks for 6 – 18 hours depending on the inherent VCD intensity of the sample. The VCD blocks are averaged, and baseline corrected with either a solvent, racemate or 'opposite' enantiomer baseline measured for the same amount of time. The IR is solvent subtracted, then both IR and VCD are compared to the DFT calculation.

The DFT calculations are completed as follows. A chosen enantiomer (either one is fine, as enantiomer VCD spectra are interconvertible through multiplication by -1) is constructed and subjected to a thorough conformer search at the molecular mechanics (MM) level. All conformers found (typically in an energy window of 5 to 7 Kcal/mol) are minimized at the DFT level – 6-31G(d) / B3LYP is a good starting point – and then the IR and VCD frequencies are calculated at the same level[9]. Unique conformers are Boltzmann averaged, and the IR / VCD spectra are plotted using a moderate line width to best match the experiment (typically 4 – 8 cm^{-1} HWHM). The spectra must be x-axis scaled to best match the experiment, which can be determined either by trial and error or using an algorithm like that found in BioTools (Jupiter, FL) CompareVOA software – the scaling factor tends to range from 0.96 to 0.99 depending on basis set and functional used in the calculation. At this point, the DFT and experimental spectra are compared[10,11] – a match will indicate that the absolute configuration of the sample is the same as the DFT calculation, while a mirror image result indicates the opposite configuration. It is best practice to use both a comparison algorithm and a visual inspection when comparing the data, and it is critical to use both the IR and VCD spectra to ensure no mistakes are made.

VCD Calculations Supporting Information

There are no imaginary frequencies in any of the conformers.

Compound (**A22153**) :

6-31G(d) / B3LYP (CPCM / Chloroform) : 3 conformers at or above 1% Boltzmann weight.

R enantiomer was calculated

Conformer # 1

Energy: -939.916608 Hartree

Center Number	Atomic Number	Atomic Type	Coordinates (Angstroms) X	Y	Z
1	6	0	-2.877155	1.344888	0.439761
2	6	0	-4.032844	0.567304	0.309480
3	6	0	-3.930312	-0.791774	-0.026800
4	6	0	-2.669413	-1.376830	-0.228705
5	6	0	-1.536318	-0.588225	-0.092800
6	6	0	-1.627952	0.767095	0.237008
7	6	0	-0.096861	-0.999793	-0.245275
8	6	0	-0.263525	1.413650	0.314902
9	7	0	0.623697	0.261206	0.009697
10	8	0	-4.996366	-1.629966	-0.179156
11	6	0	1.970822	0.330940	0.146036
12	8	0	2.587065	1.373579	0.397192
13	8	0	2.538614	-0.877650	-0.028940
14	6	0	4.004762	-1.068186	0.015360
15	6	0	4.675985	-0.240769	-1.084557
16	6	0	4.530736	-0.729522	1.413037
17	6	0	4.146670	-2.565304	-0.269823
18	6	0	-0.101875	2.608603	-0.651472
19	8	0	0.965464	3.471973	-0.313668
20	6	0	-6.305762	-1.102934	0.011203
21	1	0	1.723543	2.888216	-0.098869
22	1	0	-2.971375	2.395186	0.703011
23	1	0	-5.000645	1.026841	0.470969
24	1	0	-2.612951	-2.430628	-0.485227
25	1	0	0.132540	-1.387930	-1.245494
26	1	0	0.198333	-1.769720	0.477712
27	1	0	-0.041829	1.784835	1.326015
28	1	0	4.237709	-0.477171	-2.060339
29	1	0	4.569306	0.828706	-0.897669
30	1	0	5.742934	-0.486138	-1.122869
31	1	0	4.414844	0.332783	1.633037
32	1	0	5.593529	-0.988005	1.474777
33	1	0	3.994993	-1.310317	2.171807
34	1	0	3.623371	-3.155712	0.489317
35	1	0	3.730647	-2.813796	-1.251565
36	1	0	5.204158	-2.847721	-0.259875
37	1	0	-0.014727	2.214195	-1.677964
38	1	0	-1.016369	3.211340	-0.609397
39	1	0	-6.989302	-1.935887	-0.159990

40	1	0	-6.441442	-0.724995	1.031967
41	1	0	-6.523124	-0.300856	-0.704787

Conformer # 2

Energy: -939.916733 Hartree

Center Number	Atomic Number	Atomic Type	Coordinates (Angstroms) X	Y	Z
1	6	0	-2.855117	1.642499	0.464419
2	6	0	-4.044758	0.928586	0.372450
3	6	0	-4.034023	-0.447284	0.075488
4	6	0	-2.819684	-1.116073	-0.127234
5	6	0	-1.637562	-0.380993	-0.028686
6	6	0	-1.641324	0.980237	0.260260
7	6	0	-0.228859	-0.886258	-0.188213
8	6	0	-0.240299	1.546159	0.302562
9	7	0	0.571481	0.333782	0.020824
10	8	0	-5.260952	-1.040271	0.009015
11	6	0	1.922305	0.325109	0.135694
12	8	0	2.604380	1.334858	0.347842
13	8	0	2.413461	-0.920127	-0.012851
14	6	0	3.866025	-1.198359	0.020046
15	6	0	4.572164	-0.444517	-1.110521
16	6	0	4.429475	-0.853135	1.401320
17	6	0	3.913618	-2.708637	-0.224200
18	6	0	-0.019993	2.701260	-0.699932
19	8	0	1.101548	3.507807	-0.399062
20	6	0	-5.324004	-2.431732	-0.286882
21	1	0	1.826158	2.885278	-0.177785
22	1	0	-2.881624	2.703697	0.697229
23	1	0	-5.002815	1.414775	0.527455
24	1	0	-2.789420	-2.175758	-0.353927
25	1	0	0.029029	-1.652925	0.552623
26	1	0	-0.039115	-1.316326	-1.179840
27	1	0	0.016644	1.932456	1.299685
28	1	0	4.102192	-0.674177	-2.073077
29	1	0	4.541349	0.633907	-0.949091
30	1	0	5.619243	-0.762627	-1.159438
31	1	0	4.379029	0.220007	1.591445
32	1	0	5.475907	-1.172401	1.458296
33	1	0	3.870386	-1.379424	2.182692

34	1	0	3.365387	-3.245088	0.557045
35	1	0	3.471318	-2.958577	-1.194061
36	1	0	4.952246	-3.054001	-0.217985
37	1	0	0.030245	2.273367	-1.715405
38	1	0	-0.895977	3.359287	-0.665157
39	1	0	-6.384494	-2.687929	-0.290502
40	1	0	-4.893024	-2.651889	-1.271372
41	1	0	-4.808310	-3.027427	0.476433

Conformer # 3

Energy: -939.911694 Hartree

Center Number	Atomic Number	Atomic Type	Coordinates (Angstroms) X	Y	Z
1	6	0	2.800720	1.537984	-0.806750
2	6	0	3.970832	0.799161	-0.663555
3	6	0	3.929294	-0.521144	-0.178009
4	6	0	2.704700	-1.109687	0.166768
5	6	0	1.542530	-0.352978	0.014368
6	6	0	1.576879	0.954422	-0.466591
7	6	0	0.123404	-0.771508	0.307671
8	6	0	0.189978	1.556957	-0.507429
9	7	0	-0.643815	0.398036	-0.141127
10	8	0	5.138042	-1.146234	-0.081806
11	6	0	-1.991894	0.425911	-0.312243
12	8	0	-2.600440	1.396638	-0.756232
13	8	0	-2.542788	-0.751572	0.071313
14	6	0	-3.997381	-0.979910	0.004052
15	6	0	-4.725633	0.005996	0.923206
16	6	0	-4.478390	-0.892239	-1.447850
17	6	0	-4.132214	-2.410754	0.532001
18	6	0	0.019216	2.739058	0.467698
19	8	0	0.346584	2.405838	1.809175
20	6	0	5.170212	-2.486035	0.400020
21	1	0	1.287728	2.166103	1.824467
22	1	0	2.849529	2.555617	-1.185701
23	1	0	4.935836	1.222072	-0.924713
24	1	0	2.652479	-2.126315	0.539719
25	1	0	-0.040838	-0.974410	1.374792
26	1	0	-0.175726	-1.669561	-0.244961
27	1	0	-0.088052	1.910439	-1.509519

28	1	0	-4.326537	-0.058054	1.941515
29	1	0	-4.618082	1.031004	0.565305
30	1	0	-5.791351	-0.245613	0.958942
31	1	0	-4.369260	0.120810	-1.837526
32	1	0	-5.534243	-1.179960	-1.501846
33	1	0	-3.906093	-1.579973	-2.080307
34	1	0	-3.574473	-3.110347	-0.099275
35	1	0	-3.748176	-2.483894	1.554792
36	1	0	-5.185057	-2.710863	0.535178
37	1	0	0.622417	3.584427	0.099995
38	1	0	-1.028579	3.046427	0.472157
39	1	0	6.220879	-2.779808	0.393325
40	1	0	4.779908	-2.553420	1.422964
41	1	0	4.599703	-3.159694	-0.251146

CompareVOA results (6-31G(d) / B3LYP):

Optimal Scaling Factor = 0.970

S_{fg} (IR) = 91.5

S_{fg} (VCD S-config) = 84.7

S_{fg} (VCD R-config) = 7.6

ESI (Enantiomeric Similarity Index) = 77.1

Confidence Level = 100

Compound (**GEK**) :

6-311G(3df,2pd) / B3PW91 (CPCM / Chloroform) : 12 conformers at 1% or greater Boltzmann weight.

R,R enantiomer was calculated

Conformer # 1

Energy: -1930.410844 Hartree

Center Number	Atomic Number	Atomic Type	Coordinates (Angstroms) X	Y	Z
1	6	0	2.810541	2.788881	0.090369
2	6	0	2.848133	3.592263	1.220657
3	6	0	1.716924	4.279089	1.656075
4	6	0	0.534936	4.141601	0.924011
5	6	0	0.480355	3.346460	-0.206473
6	6	0	1.624254	2.670698	-0.621155

7	6	0	1.767654	5.164849	2.864704
8	16	0	1.551111	1.626851	-2.046283
9	8	0	2.899045	1.309119	-2.416962
10	8	0	0.653637	2.220503	-2.997821
11	7	0	0.807577	0.231434	-1.587634
12	6	0	1.321515	-0.571622	-0.472388
13	6	0	0.168691	-1.506557	-0.010718
14	7	0	-1.022266	-0.715728	0.248335
15	6	0	-2.136035	-1.095261	-0.259460
16	6	0	-4.557921	-0.869273	-0.622774
17	6	0	-3.377494	-0.391582	-0.026172
18	6	0	-3.416281	0.755958	0.787850
19	6	0	-4.639617	1.400044	0.986328
20	6	0	-5.787168	0.920041	0.394658
21	6	0	-5.760403	-0.221132	-0.418037
22	8	0	-2.317419	1.247240	1.372619
23	8	0	-6.951523	-0.600450	-0.946986
24	6	0	-6.970057	-1.743364	-1.776850
25	1	0	-0.189995	0.401729	-1.512550
26	1	0	-1.565476	0.648430	1.115430
27	6	0	0.556549	-2.284842	1.229085
28	6	0	0.709357	-1.648342	2.459740
29	6	0	1.064493	-2.373620	3.587594
30	6	0	1.272132	-3.744713	3.500815
31	6	0	1.117543	-4.385898	2.280198
32	6	0	0.759030	-3.659088	1.152217
33	6	0	4.764238	-2.941349	-1.496065
34	6	0	4.736971	-2.221288	-0.310415
35	6	0	3.630878	-1.442896	0.005421
36	6	0	2.541565	-1.376112	-0.856590
37	6	0	2.577122	-2.099845	-2.045921
38	6	0	3.680806	-2.876837	-2.363994
39	1	0	3.694563	2.265879	-0.247669
40	1	0	3.775414	3.685593	1.773424
41	1	0	-0.356790	4.666078	1.246064
42	1	0	-0.439162	3.259649	-0.769847
43	1	0	2.603187	4.905803	3.514048
44	1	0	0.845406	5.099155	3.442628
45	1	0	1.890402	6.210372	2.570001
46	1	0	1.572496	0.073067	0.377218
47	1	0	-0.019723	-2.220433	-0.822266
48	1	0	-4.496737	-1.753603	-1.242897
49	1	0	-4.668495	2.282212	1.612841
50	1	0	-6.734504	1.421154	0.549561

51	1	0	-8.001678	-1.871986	-2.094280
52	1	0	-6.646352	-2.636451	-1.234605
53	1	0	-6.336556	-1.607455	-2.658057
54	1	0	0.541327	-0.581540	2.541392
55	1	0	1.177993	-1.866462	4.537960
56	1	0	1.549375	-4.310053	4.381975
57	1	0	1.274014	-5.454886	2.202993
58	1	0	0.638611	-4.165629	0.201743
59	1	0	5.627230	-3.545861	-1.746831
60	1	0	5.579030	-2.260258	0.369761
61	1	0	3.615518	-0.880162	0.931476
62	1	0	1.743809	-2.037441	-2.734970
63	1	0	3.697790	-3.430279	-3.295052
64	1	0	-2.187857	-1.985035	-0.896925

Conformer # 2

Energy: -1930.410519 Hartree

Center Number	Atomic Number	Atomic Type	Coordinates (Angstroms) X	Y	Z
1	6	0	4.370130	-0.627010	0.357964
2	6	0	5.019937	-1.846888	0.470223
3	6	0	4.975497	-2.786452	-0.557912
4	6	0	4.265846	-2.465612	-1.717273
5	6	0	3.612604	-1.252581	-1.846014
6	6	0	3.663595	-0.336422	-0.800710
7	6	0	5.659418	-4.114005	-0.422696
8	16	0	2.810326	1.204592	-0.950192
9	8	0	2.794203	1.552577	-2.342918
10	8	0	3.361877	2.096675	0.030850
11	7	0	1.248007	0.974011	-0.547499
12	6	0	0.780492	0.842744	0.819379
13	6	0	-0.634512	1.492246	0.941989
14	7	0	-1.545676	0.843575	0.027297
15	6	0	-2.562726	0.228399	0.501536
16	6	0	-4.616168	-1.107745	0.263031
17	6	0	-3.528102	-0.449857	-0.336514
18	6	0	-3.391395	-0.453195	-1.736760
19	6	0	-4.347289	-1.117708	-2.507751
20	6	0	-5.407605	-1.758674	-1.905271
21	6	0	-5.555262	-1.760827	-0.512064

22	8	0	-2.371653	0.164449	-2.345610
23	8	0	-6.639431	-2.422520	-0.031883
24	6	0	-6.829850	-2.447723	1.367202
25	1	0	0.692147	0.561980	-1.282334
26	1	0	-1.823626	0.572503	-1.621060
27	6	0	-0.571825	2.990348	0.719933
28	6	0	-0.177589	3.805747	1.779421
29	6	0	-0.081775	5.180408	1.621023
30	6	0	-0.383354	5.761880	0.396352
31	6	0	-0.782447	4.958278	-0.662089
32	6	0	-0.876459	3.581640	-0.502239
33	6	0	0.726353	-3.123136	2.513913
34	6	0	0.526797	-2.957970	1.150252
35	6	0	0.554987	-1.688764	0.586552
36	6	0	0.783588	-0.567102	1.378022
37	6	0	0.984622	-0.743967	2.745797
38	6	0	0.957204	-2.010390	3.311970
39	1	0	4.421124	0.102174	1.154722
40	1	0	5.578097	-2.068678	1.372126
41	1	0	4.232867	-3.174718	-2.536234
42	1	0	3.079496	-1.008970	-2.755443
43	1	0	6.121751	-4.419479	-1.361887
44	1	0	4.940449	-4.890757	-0.149233
45	1	0	6.427661	-4.090093	0.349354
46	1	0	1.446166	1.455017	1.428258
47	1	0	-0.948965	1.318837	1.978960
48	1	0	-4.695754	-1.087870	1.341689
49	1	0	-4.240085	-1.118566	-3.584824
50	1	0	-6.149062	-2.273267	-2.503613
51	1	0	-7.740796	-3.015140	1.540046
52	1	0	-6.951934	-1.439061	1.772394
53	1	0	-5.996560	-2.941486	1.875454
54	1	0	0.052038	3.361607	2.742062
55	1	0	0.223511	5.798011	2.456894
56	1	0	-0.312453	6.835163	0.270111
57	1	0	-1.023799	5.402643	-1.620060
58	1	0	-1.199180	2.967004	-1.331270
59	1	0	0.707877	-4.113671	2.951318
60	1	0	0.349491	-3.819939	0.518834
61	1	0	0.406520	-1.583213	-0.480633
62	1	0	1.174523	0.119503	3.374217
63	1	0	1.123470	-2.128140	4.375825
64	1	0	-2.744749	0.198616	1.581697

Conformer # 3

Energy: -1930.410028 Hartree

Center Number	Atomic Number	Atomic Type	Coordinates (Angstroms) X	Y	Z
1	6	0	2.638436	2.927202	0.232770
2	6	0	2.588272	3.681269	1.396207
3	6	0	1.397536	4.258411	1.831403
4	6	0	0.246596	4.063217	1.063482
5	6	0	0.279347	3.316504	-0.100247
6	6	0	1.481178	2.748045	-0.512556
7	6	0	1.350610	5.088837	3.078910
8	16	0	1.522307	1.763182	-1.979726
9	8	0	2.899024	1.561676	-2.325104
10	8	0	0.606358	2.327636	-2.931403
11	7	0	0.875492	0.298047	-1.597547
12	6	0	1.420027	-0.506775	-0.498122
13	6	0	0.327961	-1.538059	-0.097535
14	7	0	-0.924355	-0.844736	0.153294
15	6	0	-1.994389	-1.289487	-0.390988
16	6	0	-4.413692	-1.222106	-0.797386
17	6	0	-3.290527	-0.684700	-0.164707
18	6	0	-3.436578	0.433423	0.686700
19	6	0	-4.705109	0.970133	0.872270
20	6	0	-5.811001	0.425955	0.237219
21	6	0	-5.673813	-0.678413	-0.606839
22	8	0	-2.388172	0.985358	1.313088
23	8	0	-6.698127	-1.275116	-1.270007
24	6	0	-7.998073	-0.749086	-1.098064
25	1	0	-0.134041	0.388146	-1.547858
26	1	0	-1.589456	0.453849	1.055763
27	6	0	0.737403	-2.325344	1.129730
28	6	0	0.805342	-1.719528	2.383208
29	6	0	1.174870	-2.453871	3.500434
30	6	0	1.482343	-3.803493	3.379985
31	6	0	1.414622	-4.413974	2.135881
32	6	0	1.041286	-3.678400	1.018454
33	6	0	5.048456	-2.582107	-1.519766
34	6	0	4.940745	-1.915224	-0.307748
35	6	0	3.774110	-1.231096	0.008646

36	6	0	2.703747	-1.207036	-0.878997
37	6	0	2.820454	-1.875991	-2.094974
38	6	0	3.984532	-2.559022	-2.413440
39	1	0	3.568476	2.490296	-0.104072
40	1	0	3.493137	3.822157	1.975404
41	1	0	-0.690188	4.503489	1.383785
42	1	0	-0.617652	3.183897	-0.690403
43	1	0	1.339059	6.153688	2.832436
44	1	0	2.217506	4.905455	3.712476
45	1	0	0.449034	4.883195	3.657115
46	1	0	1.604467	0.123134	0.379278
47	1	0	0.213095	-2.238281	-0.934212
48	1	0	-4.304004	-2.079830	-1.450437
49	1	0	-4.817546	1.826182	1.525205
50	1	0	-6.780797	0.872386	0.407200
51	1	0	-8.655207	-1.360683	-1.711140
52	1	0	-8.058417	0.289915	-1.433963
53	1	0	-8.321071	-0.810245	-0.054994
54	1	0	0.558905	-0.670424	2.490792
55	1	0	1.220796	-1.970951	4.468915
56	1	0	1.770279	-4.376208	4.252912
57	1	0	1.650547	-5.465976	2.031979
58	1	0	0.988577	-4.160742	0.049440
59	1	0	5.958741	-3.112592	-1.770823
60	1	0	5.766935	-1.921821	0.392668
61	1	0	3.696488	-0.708802	0.955076
62	1	0	2.001800	-1.844271	-2.803391
63	1	0	4.064048	-3.070511	-3.365020
64	1	0	-1.969177	-2.160680	-1.054926

Conformer # 4

Energy: -1930.409805 Hartree

Center Number	Atomic Number	Atomic Type	Coordinates (Angstroms) X	Y	Z
1	6	0	4.398861	-0.737864	0.243643
2	6	0	5.027923	-1.966717	0.382514
3	6	0	4.915374	-2.950762	-0.596710
4	6	0	4.153929	-2.668854	-1.733965
5	6	0	3.520874	-1.448987	-1.888557
6	6	0	3.643889	-0.485100	-0.892399

7	6	0	5.596856	-4.278081	-0.446959
8	16	0	2.815597	1.066028	-1.075231
9	8	0	2.732289	1.344638	-2.481124
10	8	0	3.437044	1.992635	-0.171150
11	7	0	1.272470	0.891558	-0.581226
12	6	0	0.873329	0.833965	0.811635
13	6	0	-0.499529	1.559587	0.984350
14	7	0	-1.487139	0.931686	0.136656
15	6	0	-2.498706	0.369155	0.679635
16	6	0	-4.606906	-0.886400	0.572122
17	6	0	-3.536268	-0.285085	-0.092192
18	6	0	-3.483111	-0.319305	-1.503253
19	6	0	-4.505218	-0.956030	-2.196630
20	6	0	-5.561670	-1.550319	-1.522929
21	6	0	-5.621261	-1.520238	-0.128048
22	8	0	-2.474772	0.247689	-2.180432
23	8	0	-6.615328	-2.074789	0.614127
24	6	0	-7.663526	-2.734313	-0.065525
25	1	0	0.668172	0.464921	-1.267982
26	1	0	-1.871677	0.645036	-1.497063
27	6	0	-0.374712	3.044413	0.709353
28	6	0	0.076376	3.876791	1.732269
29	6	0	0.232214	5.239319	1.523730
30	6	0	-0.065654	5.791464	0.284760
31	6	0	-0.520698	4.971017	-0.737738
32	6	0	-0.674176	3.606574	-0.527984
33	6	0	0.706954	-3.063422	2.651228
34	6	0	0.464827	-2.937257	1.290451
35	6	0	0.527403	-1.690868	0.680402
36	6	0	0.835270	-0.553921	1.421258
37	6	0	1.078724	-0.691716	2.786738
38	6	0	1.015786	-1.934924	3.399340
39	1	0	4.502841	0.025020	1.002867
40	1	0	5.621645	-2.160549	1.267923
41	1	0	4.061902	-3.416181	-2.513604
42	1	0	2.945534	-1.236797	-2.779952
43	1	0	6.148372	-4.340085	0.490096
44	1	0	6.297898	-4.452206	-1.265945
45	1	0	4.871358	-5.093965	-0.468965
46	1	0	1.599641	1.432283	1.362431
47	1	0	-0.768794	1.433563	2.040531
48	1	0	-4.651128	-0.861096	1.654521
49	1	0	-4.464284	-0.980845	-3.278085
50	1	0	-6.337783	-2.036391	-2.097341

51	1	0	-8.339055	-3.100923	0.703273
52	1	0	-7.292347	-3.580711	-0.650376
53	1	0	-8.207340	-2.051628	-0.724579
54	1	0	0.303491	3.455667	2.705790
55	1	0	0.581330	5.870490	2.331837
56	1	0	0.051745	6.855314	0.119396
57	1	0	-0.758962	5.392680	-1.706675
58	1	0	-1.038071	2.979707	-1.330259
59	1	0	0.661017	-4.036091	3.125150
60	1	0	0.226343	-3.811733	0.697554
61	1	0	0.343303	-1.616465	-0.383862
62	1	0	1.329496	0.183699	3.376048
63	1	0	1.215535	-2.022806	4.460310
64	1	0	-2.623845	0.366829	1.768068

Conformer # 5

Energy: -1930.409634 Hartree

Center Number	Atomic Number	Atomic Type	Coordinates (Angstroms) X	Y	Z
1	6	0	3.414763	-2.759596	0.823607
2	6	0	4.795021	-2.834399	0.881567
3	6	0	5.589368	-2.388641	-0.177289
4	6	0	4.955958	-1.869962	-1.304788
5	6	0	3.573642	-1.784518	-1.377707
6	6	0	2.806962	-2.228043	-0.309402
7	6	0	7.084823	-2.454759	-0.092376
8	16	0	1.043184	-2.129967	-0.388696
9	8	0	0.519824	-3.267905	0.313537
10	8	0	0.689489	-1.875364	-1.756507
11	7	0	0.547992	-0.836436	0.475291
12	6	0	0.700253	0.542007	0.020303
13	6	0	-0.590219	1.327672	0.393371
14	7	0	-1.733764	0.679437	-0.210066
15	6	0	-2.675084	0.249536	0.539343
16	6	0	-4.865242	-0.819654	0.890578
17	6	0	-3.860208	-0.389824	0.007240
18	6	0	-4.014390	-0.580313	-1.378744
19	6	0	-5.176756	-1.197245	-1.848056
20	6	0	-6.154165	-1.611153	-0.969721
21	6	0	-6.009930	-1.427169	0.411254

22	8	0	-3.082472	-0.190035	-2.254255
23	8	0	-7.037082	-1.874487	1.180507
24	6	0	-6.932985	-1.709488	2.578578
25	1	0	0.575733	-1.002129	1.471981
26	1	0	-2.347131	0.219662	-1.720531
27	6	0	-0.518575	2.770324	-0.063625
28	6	0	-0.424510	3.796120	0.871513
29	6	0	-0.358604	5.122638	0.465136
30	6	0	-0.391096	5.438244	-0.885514
31	6	0	-0.492631	4.420573	-1.826167
32	6	0	-0.556616	3.096313	-1.418587
33	6	0	4.226302	2.528377	1.507346
34	6	0	3.362150	1.892688	2.389614
35	6	0	2.230988	1.246625	1.912506
36	6	0	1.946373	1.221994	0.548058
37	6	0	2.820087	1.860828	-0.326986
38	6	0	3.952252	2.510310	0.147244
39	1	0	2.811348	-3.128141	1.642758
40	1	0	5.266447	-3.256575	1.761341
41	1	0	5.552067	-1.533028	-2.144661
42	1	0	3.089021	-1.395407	-2.262375
43	1	0	7.413382	-3.350762	0.434813
44	1	0	7.476710	-1.594321	0.456541
45	1	0	7.541144	-2.448301	-1.081695
46	1	0	0.745305	0.499422	-1.067883
47	1	0	-0.677009	1.320048	1.488638
48	1	0	-4.717383	-0.660966	1.950555
49	1	0	-5.293912	-1.343814	-2.914128
50	1	0	-7.054329	-2.088862	-1.335957
51	1	0	-6.857651	-0.653026	2.852562
52	1	0	-6.070525	-2.247917	2.982265
53	1	0	-7.843770	-2.124503	3.002970
54	1	0	-0.403095	3.556546	1.928366
55	1	0	-0.285440	5.909011	1.206459
56	1	0	-0.342697	6.471925	-1.205133
57	1	0	-0.524996	4.659039	-2.882327
58	1	0	-0.647178	2.312237	-2.160141
59	1	0	5.109206	3.033390	1.879135
60	1	0	3.567630	1.901189	3.453006
61	1	0	1.568408	0.761429	2.620553
62	1	0	2.612843	1.850723	-1.390445
63	1	0	4.621239	3.000689	-0.549224
64	1	0	-2.621552	0.356313	1.629069

Conformer # 6

Energy: -1930.409518 Hartree

Center Number	Atomic Number	Atomic Type	Coordinates (Angstroms) X	Y	Z
1	6	0	-4.335619	1.244275	0.283479
2	6	0	-5.431581	0.654312	0.897379
3	6	0	-5.951443	-0.553870	0.440204
4	6	0	-5.340653	-1.164767	-0.658684
5	6	0	-4.245029	-0.593569	-1.279164
6	6	0	-3.743782	0.614777	-0.803053
7	6	0	-7.147482	-1.180712	1.091925
8	16	0	-2.342971	1.349228	-1.592571
9	8	0	-2.310773	2.729236	-1.202705
10	8	0	-2.370119	0.972791	-2.977629
11	7	0	-0.994414	0.645740	-0.984164
12	6	0	-0.614186	0.817146	0.415990
13	6	0	0.170516	-0.444994	0.894847
14	7	0	1.347079	-0.633708	0.081380
15	6	0	2.498505	-0.584930	0.636331
16	6	0	4.956485	-0.706275	0.579769
17	6	0	3.729584	-0.754280	-0.105066
18	6	0	3.710350	-0.964116	-1.496412
19	6	0	4.921918	-1.124448	-2.172218
20	6	0	6.115776	-1.077036	-1.486801
21	6	0	6.147559	-0.866338	-0.101971
22	8	0	2.565158	-1.012290	-2.187077
23	8	0	7.381447	-0.837195	0.464358
24	6	0	7.462376	-0.619716	1.857297
25	1	0	-0.858346	-0.282088	-1.364203
26	1	0	1.834128	-0.868741	-1.526659
27	6	0	-0.714195	-1.679010	0.894767
28	6	0	-0.611275	-2.670038	-0.077534
29	6	0	-1.449164	-3.778952	-0.047897
30	6	0	-2.394237	-3.915213	0.957323
31	6	0	-2.497435	-2.935263	1.937611
32	6	0	-1.664237	-1.827679	1.905184
33	6	0	1.723415	4.341405	1.222911
34	6	0	1.816953	3.721725	-0.016341
35	6	0	1.054477	2.596283	-0.296668

36	6	0	0.187238	2.076857	0.659546
37	6	0	0.095776	2.705705	1.898538
38	6	0	0.858129	3.830744	2.181044
39	1	0	-3.953609	2.195319	0.628585
40	1	0	-5.894371	1.148937	1.743064
41	1	0	-5.734023	-2.101489	-1.035904
42	1	0	-3.789690	-1.069398	-2.137512
43	1	0	-7.426275	-0.654906	2.004070
44	1	0	-6.955628	-2.225389	1.342633
45	1	0	-8.007951	-1.166055	0.418882
46	1	0	-1.509538	0.854390	1.045727
47	1	0	0.449985	-0.230520	1.933999
48	1	0	4.940492	-0.541303	1.648823
49	1	0	4.904435	-1.285722	-3.242386
50	1	0	7.054797	-1.201566	-2.011502
51	1	0	8.520662	-0.628491	2.105585
52	1	0	6.955285	-1.411728	2.416004
53	1	0	7.035253	0.347884	2.136185
54	1	0	0.141233	-2.589973	-0.851178
55	1	0	-1.353603	-4.540998	-0.811637
56	1	0	-3.041746	-4.782782	0.984211
57	1	0	-3.223365	-3.037498	2.734815
58	1	0	-1.745359	-1.074857	2.681508
59	1	0	2.315816	5.222321	1.437743
60	1	0	2.484940	4.117564	-0.771722
61	1	0	1.120399	2.123574	-1.267648
62	1	0	-0.586309	2.318836	2.648198
63	1	0	0.769117	4.312978	3.146925
64	1	0	2.594025	-0.412654	1.714422

Conformer # 7

Energy: -1930.409149 Hartree

Center Number	Atomic Number	Atomic Type	Coordinates (Angstroms) X	Y	Z
1	6	0	-3.556230	-2.187954	-0.895758
2	6	0	-4.488839	-2.825477	-0.092145
3	6	0	-5.631906	-2.163836	0.353146
4	6	0	-5.824359	-0.837843	-0.039965
5	6	0	-4.904125	-0.186712	-0.842550
6	6	0	-3.766471	-0.866242	-1.265020

7	6	0	-6.623085	-2.850357	1.244281
8	16	0	-2.581365	-0.034406	-2.277978
9	8	0	-3.289890	0.932442	-3.068468
10	8	0	-1.763986	-1.040034	-2.897129
11	7	0	-1.595996	0.843951	-1.318448
12	6	0	-0.559434	0.252344	-0.486272
13	6	0	0.720998	1.149079	-0.615161
14	7	0	1.825063	0.494198	0.048350
15	6	0	2.838042	0.143489	-0.649094
16	6	0	5.070291	-0.864988	-0.897853
17	6	0	3.987895	-0.519087	-0.070614
18	6	0	4.030769	-0.817901	1.303957
19	6	0	5.160913	-1.456608	1.819793
20	6	0	6.214407	-1.788575	0.996308
21	6	0	6.181617	-1.496946	-0.373433
22	8	0	3.021038	-0.510119	2.125739
23	8	0	7.275588	-1.871389	-1.086042
24	6	0	7.285310	-1.596982	-2.471238
25	1	0	-2.004315	1.718462	-1.019589
26	1	0	2.324356	-0.068366	1.565207
27	6	0	0.521676	2.558392	-0.099112
28	6	0	0.740023	2.895628	1.236016
29	6	0	0.536385	4.195481	1.677371
30	6	0	0.109000	5.178127	0.793778
31	6	0	-0.104892	4.854569	-0.538675
32	6	0	0.105702	3.555122	-0.981394
33	6	0	-1.683695	-0.643069	3.579502
34	6	0	-2.180279	0.497657	2.963855
35	6	0	-1.829416	0.794418	1.653694
36	6	0	-0.979676	-0.045172	0.936968
37	6	0	-0.494352	-1.191949	1.563064
38	6	0	-0.839134	-1.489214	2.873971
39	1	0	-2.680406	-2.714138	-1.249616
40	1	0	-4.327565	-3.859829	0.187662
41	1	0	-6.714820	-0.310061	0.280865
42	1	0	-5.073493	0.835336	-1.154511
43	1	0	-6.415161	-2.625310	2.293760
44	1	0	-6.581351	-3.932785	1.127143
45	1	0	-7.640520	-2.517869	1.037579
46	1	0	-0.292252	-0.688827	-0.967673
47	1	0	0.906370	1.211813	-1.694444
48	1	0	5.008055	-0.622968	-1.950489
49	1	0	5.192687	-1.685496	2.877293
50	1	0	7.089691	-2.283734	1.397943

51	1	0	8.233645	-1.970853	-2.848873
52	1	0	7.218635	-0.522825	-2.666845
53	1	0	6.466594	-2.107982	-2.986140
54	1	0	1.082254	2.141137	1.930941
55	1	0	0.715101	4.441473	2.717058
56	1	0	-0.047198	6.192262	1.140410
57	1	0	-0.425873	5.615226	-1.239804
58	1	0	-0.044340	3.313843	-2.027336
59	1	0	-1.958471	-0.874249	4.601266
60	1	0	-2.844044	1.161722	3.503807
61	1	0	-2.231444	1.689963	1.196708
62	1	0	0.155834	-1.863403	1.015196
63	1	0	-0.451343	-2.386037	3.341143
64	1	0	2.872931	0.337397	-1.727635

Conformer # 8

Energy: -1930.409106 Hartree

Center Number	Atomic Number	Atomic Type	Coordinates (Angstroms) X	Y	Z
1	6	0	3.094520	-2.822574	0.539198
2	6	0	4.293972	-3.072739	1.179617
3	6	0	5.482092	-2.474195	0.750691
4	6	0	5.431216	-1.611839	-0.341796
5	6	0	4.235797	-1.349108	-0.995169
6	6	0	3.072258	-1.955571	-0.548281
7	6	0	6.780282	-2.776892	1.437065
8	16	0	1.550611	-1.664387	-1.390305
9	8	0	0.852669	-2.917144	-1.505977
10	8	0	1.847517	-0.896036	-2.569765
11	7	0	0.642050	-0.774687	-0.337866
12	6	0	0.645092	0.690167	-0.430235
13	6	0	-0.623682	1.207364	0.298736
14	7	0	-1.792717	0.537559	-0.243093
15	6	0	-2.654225	0.041541	0.565305
16	6	0	-4.765830	-1.121987	1.055715
17	6	0	-3.865565	-0.602091	0.108464
18	6	0	-4.153783	-0.705273	-1.265865
19	6	0	-5.341841	-1.326383	-1.659537
20	6	0	-6.213562	-1.829579	-0.719611
21	6	0	-5.935952	-1.733742	0.650584

22	8	0	-3.326409	-0.229172	-2.201523
23	8	0	-6.868341	-2.265152	1.482579
24	6	0	-6.628359	-2.193894	2.872372
25	1	0	-0.293503	-1.168739	-0.334635
26	1	0	-2.552586	0.171256	-1.720643
27	6	0	-0.763492	2.707974	0.143209
28	6	0	-0.561047	3.543827	1.236792
29	6	0	-0.681327	4.921486	1.106487
30	6	0	-1.010571	5.477817	-0.121154
31	6	0	-1.220500	4.649407	-1.216779
32	6	0	-1.097842	3.273926	-1.085887
33	6	0	4.172409	2.462274	1.284007
34	6	0	3.733094	2.853022	0.027080
35	6	0	2.602885	2.267044	-0.528329
36	6	0	1.899114	1.287877	0.164943
37	6	0	2.349709	0.897171	1.424227
38	6	0	3.477933	1.480620	1.980934
39	1	0	2.182627	-3.298420	0.875335
40	1	0	4.312141	-3.747201	2.027687
41	1	0	6.340274	-1.135846	-0.689714
42	1	0	4.201944	-0.682481	-1.845520
43	1	0	6.649636	-2.853685	2.516957
44	1	0	7.527386	-2.011000	1.232136
45	1	0	7.183684	-3.732669	1.092271
46	1	0	0.570569	0.996494	-1.476949
47	1	0	-0.515369	0.978616	1.366332
48	1	0	-4.516869	-1.027853	2.104309
49	1	0	-5.562578	-1.404790	-2.716267
50	1	0	-7.133487	-2.310892	-1.027264
51	1	0	-7.479548	-2.669496	3.352940
52	1	0	-6.555800	-1.156992	3.213163
53	1	0	-5.714575	-2.728176	3.148194
54	1	0	-0.305036	3.114286	2.198236
55	1	0	-0.519038	5.558750	1.967177
56	1	0	-1.107160	6.551587	-0.224329
57	1	0	-1.482883	5.075723	-2.177335
58	1	0	-1.274651	2.636090	-1.942895
59	1	0	5.054845	2.916293	1.717914
60	1	0	4.271740	3.612855	-0.525801
61	1	0	2.265994	2.570589	-1.512472
62	1	0	1.821451	0.120824	1.964100
63	1	0	3.818724	1.166189	2.959888
64	1	0	-2.503287	0.094924	1.649576

Conformer # 9

Energy: -1930.409003 Hartree

Center Number	Atomic Number	Atomic Type	Coordinates (Angstroms) X	Y	Z
1	6	0	-3.394323	-2.817901	-0.828898
2	6	0	-4.771962	-2.932318	-0.858834
3	6	0	-5.555870	-2.531210	0.226443
4	6	0	-4.914142	-2.009255	1.346678
5	6	0	-3.532865	-1.883839	1.391589
6	6	0	-2.777383	-2.288766	0.301176
7	6	0	-7.047214	-2.680116	0.184215
8	16	0	-1.016467	-2.134839	0.341234
9	8	0	-0.474441	-3.249940	-0.383001
10	8	0	-0.638591	-1.880455	1.702603
11	7	0	-0.583521	-0.818698	-0.522428
12	6	0	-0.762584	0.549971	-0.047372
13	6	0	0.496256	1.376451	-0.438671
14	7	0	1.673488	0.751049	0.123859
15	6	0	2.602906	0.359690	-0.658835
16	6	0	4.808066	-0.636343	-1.081791
17	6	0	3.822737	-0.253103	-0.170628
18	6	0	4.029576	-0.462242	1.211368
19	6	0	5.220456	-1.047675	1.626321
20	6	0	6.191405	-1.421783	0.709366
21	6	0	5.992182	-1.218175	-0.657427
22	8	0	3.111702	-0.112957	2.121187
23	8	0	6.885596	-1.551264	-1.626995
24	6	0	8.102906	-2.147767	-1.231066
25	1	0	-0.639005	-0.973995	-1.519704
26	1	0	2.350465	0.283232	1.618040
27	6	0	0.397767	2.808433	0.045502
28	6	0	0.271663	3.848388	-0.869961
29	6	0	0.181791	5.165656	-0.438906
30	6	0	0.222178	5.457765	0.916821
31	6	0	0.355724	4.425898	1.837836
32	6	0	0.443761	3.110860	1.405659
33	6	0	-4.375568	2.447365	-1.438974
34	6	0	-3.505245	1.858018	-2.346974
35	6	0	-2.345148	1.241911	-1.900777

36	6	0	-2.037379	1.201243	-0.541695
37	6	0	-2.917039	1.794239	0.359248
38	6	0	-4.078386	2.413412	-0.084084
39	1	0	-2.798759	-3.149185	-1.669510
40	1	0	-5.250325	-3.346563	-1.738754
41	1	0	-5.502083	-1.697331	2.201747
42	1	0	-3.040881	-1.488987	2.269631
43	1	0	-7.332392	-3.734588	0.211277
44	1	0	-7.458406	-2.261118	-0.735689
45	1	0	-7.521653	-2.183439	1.029408
46	1	0	-0.784656	0.493305	1.040823
47	1	0	0.555319	1.389816	-1.535613
48	1	0	4.651004	-0.476944	-2.142163
49	1	0	5.379271	-1.208706	2.684998
50	1	0	7.103841	-1.873979	1.072345
51	1	0	8.662492	-2.329743	-2.145263
52	1	0	7.935935	-3.098903	-0.717541
53	1	0	8.682538	-1.485308	-0.581849
54	1	0	0.244417	3.627367	-1.930712
55	1	0	0.083698	5.963377	-1.165073
56	1	0	0.154938	6.484284	1.255519
57	1	0	0.394367	4.646079	2.897739
58	1	0	0.559153	2.315793	2.131971
59	1	0	-5.281112	2.928713	-1.786922
60	1	0	-3.728572	1.879476	-3.406588
61	1	0	-1.678443	0.792769	-2.628583
62	1	0	-2.691626	1.771916	1.418826
63	1	0	-4.751702	2.867734	0.632379
64	1	0	2.516149	0.479973	-1.744834

Conformer # 10

Energy: -1930.408758 Hartree

Center Number	Atomic Number	Atomic Type	Coordinates (Angstroms) X	Y	Z
1	6	0	-4.397754	1.245125	0.168198
2	6	0	-5.509858	0.672099	0.768832
3	6	0	-6.004668	-0.558963	0.345657
4	6	0	-5.350926	-1.210784	-0.703676
5	6	0	-4.239701	-0.655917	-1.311290
6	6	0	-3.764764	0.576222	-0.870774

7	6	0	-7.221608	-1.165254	0.977717
8	16	0	-2.344608	1.290966	-1.643610
9	8	0	-2.327417	2.682177	-1.294887
10	8	0	-2.331302	0.873587	-3.017237
11	7	0	-1.011625	0.608787	-0.978086
12	6	0	-0.685507	0.799266	0.433122
13	6	0	0.105525	-0.443752	0.949620
14	7	0	1.317104	-0.611767	0.183930
15	6	0	2.441500	-0.541741	0.787085
16	6	0	4.895132	-0.621753	0.828927
17	6	0	3.707526	-0.687273	0.097728
18	6	0	3.758163	-0.890978	-1.299114
19	6	0	4.998833	-1.022827	-1.911294
20	6	0	6.170060	-0.957437	-1.171759
21	6	0	6.127600	-0.755442	0.209435
22	8	0	2.643766	-0.956682	-2.040594
23	8	0	7.221253	-0.677368	1.012270
24	6	0	8.494858	-0.804442	0.414577
25	1	0	-0.859905	-0.324625	-1.338451
26	1	0	1.882457	-0.831788	-1.414510
27	6	0	-0.753103	-1.695625	0.923901
28	6	0	-0.597527	-2.690149	-0.037915
29	6	0	-1.413923	-3.815531	-0.028563
30	6	0	-2.389055	-3.964855	0.945743
31	6	0	-2.545185	-2.980898	1.914880
32	6	0	-1.734409	-1.856598	1.901966
33	6	0	1.552788	4.374277	1.295322
34	6	0	1.701209	3.748919	0.064240
35	6	0	0.971743	2.606376	-0.234083
36	6	0	0.082580	2.075906	0.695331
37	6	0	-0.063862	2.710169	1.926337
38	6	0	0.666108	3.851815	2.227175
39	1	0	-4.033792	2.212258	0.487079
40	1	0	-6.004636	1.197337	1.577190
41	1	0	-5.723229	-2.166922	-1.052346
42	1	0	-3.751778	-1.163919	-2.132490
43	1	0	-8.076790	-1.110442	0.299573
44	1	0	-7.492615	-0.649381	1.897877
45	1	0	-7.062479	-2.219860	1.207377
46	1	0	-1.603412	0.824443	1.030300
47	1	0	0.340380	-0.218413	1.997394
48	1	0	4.859030	-0.464196	1.900414
49	1	0	5.037259	-1.177882	-2.981935
50	1	0	7.115820	-1.064906	-1.684226

51	1	0	9.218064	-0.712030	1.220965
52	1	0	8.674159	-0.014397	-0.320156
53	1	0	8.617170	-1.778858	-0.066790
54	1	0	0.179093	-2.599378	-0.786166
55	1	0	-1.277379	-4.580287	-0.783302
56	1	0	-3.019115	-4.845501	0.957018
57	1	0	-3.295588	-3.092851	2.687727
58	1	0	-1.857106	-1.100569	2.669614
59	1	0	2.119779	5.268193	1.524438
60	1	0	2.386669	4.153595	-0.670482
61	1	0	1.080183	2.128694	-1.198691
62	1	0	-0.763231	2.313566	2.654707
63	1	0	0.535220	4.337445	3.186552
64	1	0	2.490096	-0.367366	1.867803

Conformer # 11

Energy: -1930.408483 Hartree

Center Number	Atomic Number	Atomic Type	Coordinates (Angstroms) X	Y	Z
1	6	0	-3.549321	-2.237946	-0.921632
2	6	0	-4.433414	-2.936088	-0.113431
3	6	0	-5.581484	-2.330279	0.393846
4	6	0	-5.829630	-0.997564	0.058649
5	6	0	-4.958471	-0.286402	-0.747606
6	6	0	-3.814310	-0.911168	-1.232635
7	6	0	-6.519913	-3.082107	1.289352
8	16	0	-2.691234	-0.002673	-2.250299
9	8	0	-3.456789	0.965692	-2.983608
10	8	0	-1.859521	-0.956215	-2.929734
11	7	0	-1.706939	0.875885	-1.289295
12	6	0	-0.628712	0.289466	-0.507643
13	6	0	0.625413	1.219626	-0.655171
14	7	0	1.766610	0.570674	-0.050414
15	6	0	2.763688	0.271346	-0.791141
16	6	0	5.008350	-0.666225	-1.134473
17	6	0	3.949150	-0.381185	-0.271044
18	6	0	4.047333	-0.731550	1.094162
19	6	0	5.206668	-1.353942	1.542254
20	6	0	6.251466	-1.629851	0.672955
21	6	0	6.160224	-1.287169	-0.677579

22	8	0	3.054357	-0.481400	1.957921
23	8	0	7.131089	-1.518415	-1.600303
24	6	0	8.319246	-2.149585	-1.170353
25	1	0	-2.137039	1.723008	-0.945473
26	1	0	2.330474	-0.038537	1.437414
27	6	0	0.414514	2.608114	-0.089875
28	6	0	0.679766	2.912411	1.244639
29	6	0	0.463871	4.193710	1.732111
30	6	0	-0.022879	5.190179	0.896058
31	6	0	-0.283766	4.899520	-0.435623
32	6	0	-0.060798	3.618985	-0.924831
33	6	0	-1.596064	-0.733248	3.568529
34	6	0	-2.132358	0.414613	3.001395
35	6	0	-1.832085	0.752068	1.688561
36	6	0	-0.994212	-0.053440	0.920432
37	6	0	-0.468339	-1.207534	1.498438
38	6	0	-0.762459	-1.545378	2.811898
39	1	0	-2.669498	-2.720980	-1.323761
40	1	0	-4.229456	-3.974236	0.120737
41	1	0	-6.725270	-0.512238	0.428383
42	1	0	-5.171037	0.740407	-1.014219
43	1	0	-6.292007	-2.880292	2.339395
44	1	0	-6.439648	-4.158031	1.137640
45	1	0	-7.554873	-2.784175	1.120149
46	1	0	-0.355680	-0.632534	-1.021804
47	1	0	0.770690	1.318090	-1.737820
48	1	0	4.935001	-0.398895	-2.182101
49	1	0	5.282121	-1.622220	2.588326
50	1	0	7.136635	-2.114884	1.060086
51	1	0	8.950862	-2.237745	-2.050780
52	1	0	8.121389	-3.147830	-0.769620
53	1	0	8.839196	-1.554479	-0.414225
54	1	0	1.068210	2.147217	1.902580
55	1	0	0.679601	4.414291	2.770479
56	1	0	-0.188633	6.189819	1.278496
57	1	0	-0.651275	5.671670	-1.100406
58	1	0	-0.247867	3.404059	-1.970519
59	1	0	-1.831422	-0.996158	4.592456
60	1	0	-2.787618	1.052642	3.581615
61	1	0	-2.263817	1.652986	1.270848
62	1	0	0.173855	-1.852599	0.910893
63	1	0	-0.343738	-2.447266	3.241069
64	1	0	2.760956	0.502215	-1.862748

Conformer # 12

Energy: -1930.408478

Center Number	Atomic Number	Atomic Type	Coordinates (Angstroms) X	Y	Z
1	6	0	-3.065221	-2.890882	-0.566554
2	6	0	-4.265500	-3.172873	-1.192281
3	6	0	-5.465277	-2.615559	-0.741215
4	6	0	-5.425405	-1.760763	0.357800
5	6	0	-4.229542	-1.466397	0.996459
6	6	0	-3.054273	-2.032977	0.528143
7	6	0	-6.763626	-2.953068	-1.410831
8	16	0	-1.530047	-1.702020	1.350933
9	8	0	-0.791439	-2.933264	1.442116
10	8	0	-1.833716	-0.955956	2.542906
11	7	0	-0.665646	-0.772096	0.296130
12	6	0	-0.706725	0.690664	0.410101
13	6	0	0.531503	1.253695	-0.336354
14	7	0	1.730519	0.604664	0.165463
15	6	0	2.587269	0.155903	-0.672880
16	6	0	4.718742	-0.926867	-1.228220
17	6	0	3.827558	-0.464611	-0.257192
18	6	0	4.151850	-0.601998	1.111590
19	6	0	5.360282	-1.198368	1.453964
20	6	0	6.235009	-1.651956	0.478768
21	6	0	5.920470	-1.519215	-0.875699
22	8	0	3.329352	-0.176246	2.078174
23	8	0	6.715696	-1.933032	-1.897009
24	6	0	7.948637	-2.542531	-1.574236
25	1	0	0.279445	-1.141305	0.267420
26	1	0	2.537022	0.216895	1.626299
27	6	0	0.639012	2.753864	-0.154629
28	6	0	0.974199	3.304890	1.081085
29	6	0	1.069912	4.680117	1.235028
30	6	0	0.831339	5.523006	0.156437
31	6	0	0.500845	4.981643	-1.077520
32	6	0	0.407998	3.604288	-1.231147
33	6	0	-4.313769	2.388079	-1.211334
34	6	0	-3.604749	1.437704	-1.936369
35	6	0	-2.450217	0.878240	-1.409761

36	6	0	-1.987574	1.262306	-0.152818
37	6	0	-2.705234	2.210788	0.568256
38	6	0	-3.862108	2.772339	0.043205
39	1	0	-2.143897	-3.335100	-0.919810
40	1	0	-4.274857	-3.840143	-2.046143
41	1	0	-6.343664	-1.315903	0.722427
42	1	0	-4.203935	-0.805305	1.851379
43	1	0	-6.641706	-3.043914	-2.490544
44	1	0	-7.523238	-2.198368	-1.210059
45	1	0	-7.146050	-3.910631	-1.047379
46	1	0	-0.620651	0.984172	1.459601
47	1	0	0.404513	1.042586	-1.405385
48	1	0	4.471768	-0.821743	-2.278091
49	1	0	5.609063	-1.304146	2.502152
50	1	0	7.164521	-2.110574	0.786051
51	1	0	8.417975	-2.795849	-2.521532
52	1	0	7.804234	-3.455707	-0.990055
53	1	0	8.601335	-1.860582	-1.021839
54	1	0	1.172883	2.655947	1.924929
55	1	0	1.333507	5.094974	2.200256
56	1	0	0.906676	6.596582	0.277731
57	1	0	0.316340	5.630409	-1.925028
58	1	0	0.151784	3.186470	-2.197681
59	1	0	-5.216871	2.822882	-1.621771
60	1	0	-3.954815	1.128713	-2.913786
61	1	0	-1.910341	0.125749	-1.971438
62	1	0	-2.357895	2.509366	1.550299
63	1	0	-4.411507	3.507962	0.617794
64	1	0	2.413438	0.232122	-1.752136

CompareVOA results (6-311G(3df,2pd) / B3PW91):

Optimal Scaling Factor = 0.978

S_{fg} (IR) = 87.8

S_{fg} (VCD R,R-config) = 75.1

S_{fg} (VCD S,S-config) = 15.8

ESI (Enantiomeric Similarity Index) = 59.2

Confidence Level = 99

Compound (**JQ1**) :

cc-pVTZ / B3PW91 (CPCM / Chloroform) : 6 conformers used in Boltzmann average.

S enantiomer was calculated

Conformer # 1

Energy: -2119.277312 Hartree

Center Number	Atomic Number	Atomic Type	Coordinates (Angstroms) X	Y	Z
1	16	0	2.575949	-3.052609	1.089001
2	6	0	2.849859	-1.762091	2.208050
3	6	0	2.189270	-0.622414	1.841291
4	6	0	1.453175	-0.783857	0.618239
5	6	0	1.570505	-2.057115	0.109728
6	7	0	-0.489507	0.079612	-0.584960
7	7	0	0.888592	-2.535883	-1.007157
8	6	0	-0.412903	-2.195702	-1.300292
9	6	0	-1.135232	-1.223110	-0.432577
10	7	0	-0.794583	-2.789179	-2.391213
11	6	0	1.261659	-3.395937	-2.014895
12	7	0	0.260598	-3.549640	-2.839466
13	6	0	3.723252	-1.991150	3.397115
14	6	0	2.592089	-4.030966	-2.151461
15	6	0	0.671960	0.261198	-0.079341
16	6	0	2.669414	1.732897	-0.445872
17	6	0	1.292701	1.596661	-0.273373
18	6	0	0.490452	2.736407	-0.342688
19	6	0	1.045076	3.983684	-0.570272
20	6	0	2.418535	4.090414	-0.743219
21	6	0	3.238147	2.974025	-0.688132
22	17	0	3.124117	5.655615	-1.032301
23	6	0	2.199748	0.624727	2.670596
24	6	0	-2.603486	-1.118508	-0.797611
25	6	0	-3.389542	-0.341191	0.232783
26	8	0	-3.022859	-0.159122	1.370722
27	8	0	-4.543937	0.072911	-0.287401
28	6	0	-5.555360	0.794004	0.504449
29	6	0	-6.670127	1.020033	-0.505186
30	6	0	-4.988962	2.122526	0.984478
31	6	0	-6.041052	-0.080308	1.651543
32	1	0	-1.046835	-1.564776	0.607199
33	1	0	4.651618	-1.419662	3.325058
34	1	0	3.987445	-3.042627	3.504520
35	1	0	3.217559	-1.682101	4.314390

36	1	0	2.764668	-4.785239	-1.381405
37	1	0	2.639151	-4.517767	-3.123203
38	1	0	3.394404	-3.294233	-2.084964
39	1	0	3.307807	0.859131	-0.408646
40	1	0	-0.578490	2.635035	-0.212297
41	1	0	0.421743	4.866491	-0.612112
42	1	0	4.305026	3.073208	-0.834126
43	1	0	2.371520	0.387862	3.720545
44	1	0	1.250520	1.155011	2.597118
45	1	0	2.984427	1.317168	2.357406
46	1	0	-2.725238	-0.659202	-1.777484
47	1	0	-3.047999	-2.115162	-0.860835
48	1	0	-6.311026	1.608513	-1.350291
49	1	0	-7.049260	0.068612	-0.880198
50	1	0	-7.492986	1.558420	-0.033940
51	1	0	-4.593007	2.693494	0.143065
52	1	0	-4.198461	1.977822	1.717075
53	1	0	-5.787758	2.707066	1.444063
54	1	0	-5.258959	-0.243951	2.389269
55	1	0	-6.884189	0.409211	2.141807
56	1	0	-6.383265	-1.046081	1.275989

Conformer # 2

Energy: -2119.276876 Hartree

Center Number	Atomic Number	Atomic Type	Coordinates (Angstroms) X	Y	Z
1	16	0	0.702398	3.568371	-0.364371
2	6	0	1.858274	3.056011	-1.543981
3	6	0	2.047894	1.702065	-1.515782
4	6	0	1.253539	1.057236	-0.507240
5	6	0	0.471459	1.954470	0.184463
6	7	0	0.202892	-1.083557	0.032476
7	7	0	-0.472191	1.610625	1.150095
8	6	0	-1.266131	0.491214	1.059386
9	6	0	-1.105902	-0.445245	-0.089395
10	7	0	-2.042954	0.408335	2.096826
11	6	0	-0.841785	2.207564	2.334490
12	7	0	-1.778364	1.493886	2.899383
13	6	0	2.503574	4.067937	-2.432097
14	6	0	-0.258140	3.451143	2.886564

15	6	0	1.255133	-0.386882	-0.182657
16	6	0	3.678242	-0.398465	0.462418
17	6	0	2.562862	-1.075044	-0.028978
18	6	0	2.676730	-2.434891	-0.321574
19	6	0	3.873965	-3.104054	-0.137704
20	6	0	4.967997	-2.407017	0.357383
21	6	0	4.880795	-1.058360	0.664823
22	17	0	6.477115	-3.241999	0.594359
23	6	0	2.948110	0.991104	-2.478882
24	6	0	-2.190493	-1.508353	-0.114840
25	6	0	-3.542115	-0.967583	-0.517297
26	8	0	-3.708457	0.087672	-1.084796
27	8	0	-4.498830	-1.833282	-0.188621
28	6	0	-5.920340	-1.594325	-0.493112
29	6	0	-6.122883	-1.503496	-1.998657
30	6	0	-6.405289	-0.350210	0.237058
31	6	0	-6.594240	-2.838865	0.063920
32	1	0	-1.159759	0.148084	-1.011403
33	1	0	2.394790	3.787906	-3.482121
34	1	0	2.058637	5.054387	-2.306449
35	1	0	3.572503	4.155147	-2.224048
36	1	0	0.831924	3.404768	2.908920
37	1	0	-0.627062	3.579414	3.901888
38	1	0	-0.545243	4.328952	2.304384
39	1	0	3.606845	0.653611	0.708229
40	1	0	1.812979	-2.965806	-0.698657
41	1	0	3.961774	-4.155359	-0.375937
42	1	0	5.739660	-0.532172	1.058290
43	1	0	3.961704	0.881908	-2.086266
44	1	0	3.018466	1.541009	-3.417492
45	1	0	2.577930	-0.008882	-2.703503
46	1	0	-2.268752	-2.010102	0.848659
47	1	0	-1.911247	-2.271054	-0.846211
48	1	0	-7.191702	-1.457998	-2.214196
49	1	0	-5.716476	-2.388041	-2.492040
50	1	0	-5.647726	-0.616875	-2.411642
51	1	0	-5.942981	0.551249	-0.158081
52	1	0	-7.487095	-0.266466	0.120817
53	1	0	-6.183597	-0.423779	1.302837
54	1	0	-7.670496	-2.782859	-0.102464
55	1	0	-6.414479	-2.927207	1.136005
56	1	0	-6.214844	-3.735652	-0.427654

Conformer # 3

Energy: -2119.275182 Hartree

Center Number	Atomic Number	Atomic Type	Coordinates (Angstroms) X	Y	Z
1	16	0	-0.499454	3.562299	1.440172
2	6	0	-1.128289	2.385981	2.540454
3	6	0	-1.144855	1.133599	1.991560
4	6	0	-0.650754	1.118728	0.642376
5	6	0	-0.260915	2.370430	0.222942
6	7	0	0.423973	-0.336425	-1.000655
7	7	0	0.341365	2.651598	-1.001468
8	6	0	1.259355	1.819419	-1.600951
9	6	0	1.608535	0.518277	-0.961010
10	7	0	1.654674	2.329171	-2.728502
11	6	0	0.218435	3.717297	-1.864001
12	7	0	1.000788	3.528193	-2.892475
13	6	0	-1.572105	2.812700	3.900462
14	6	0	-0.672566	4.882771	-1.665018
15	6	0	-0.576477	-0.057843	-0.252191
16	6	0	-3.053707	-0.443441	-0.205210
17	6	0	-1.762103	-0.946855	-0.352918
18	6	0	-1.598682	-2.297576	-0.664282
19	6	0	-2.693743	-3.129952	-0.814829
20	6	0	-3.969227	-2.601821	-0.665644
21	6	0	-4.161087	-1.262055	-0.366842
22	17	0	-5.351933	-3.641856	-0.856867
23	6	0	-1.587799	-0.076916	2.754678
24	6	0	2.761681	-0.163384	-1.682970
25	6	0	3.207421	-1.460525	-1.046749
26	8	0	3.422237	-2.468655	-1.676889
27	8	0	3.367742	-1.320963	0.270646
28	6	0	3.858731	-2.413449	1.127936
29	6	0	2.890048	-3.586558	1.088477
30	6	0	3.871998	-1.776660	2.509125
31	6	0	5.267094	-2.809104	0.707874
32	1	0	1.893303	0.722486	0.079162
33	1	0	-1.116407	2.187413	4.671093
34	1	0	-2.656179	2.729885	4.008774
35	1	0	-1.295301	3.845871	4.107369
36	1	0	-0.686204	5.462359	-2.585569

37	1	0	-1.690838	4.570260	-1.427715
38	1	0	-0.323071	5.529802	-0.858104
39	1	0	-3.203138	0.604976	0.020246
40	1	0	-0.598906	-2.691140	-0.789199
41	1	0	-2.563060	-4.178354	-1.046153
42	1	0	-5.161353	-0.864050	-0.264195
43	1	0	-1.397005	0.048819	3.820698
44	1	0	-2.657101	-0.265549	2.633532
45	1	0	-1.061436	-0.971181	2.422768
46	1	0	2.486747	-0.378126	-2.713024
47	1	0	3.623145	0.509015	-1.700068
48	1	0	1.881648	-3.257694	1.343967
49	1	0	2.872230	-4.055861	0.107796
50	1	0	3.200053	-4.329882	1.824861
51	1	0	2.870265	-1.453260	2.794717
52	1	0	4.536019	-0.911532	2.529531
53	1	0	4.224088	-2.499433	3.245872
54	1	0	5.921362	-1.935793	0.695126
55	1	0	5.668526	-3.521347	1.430638
56	1	0	5.275361	-3.272103	-0.276110

Conformer # 4

Energy: -2119.274800 Hartree

Center Number	Atomic Number	Atomic Type	Coordinates (Angstroms) X	Y	Z
1	16	0	-0.246443	3.276521	-0.032216
2	6	0	0.916280	3.126165	-1.303234
3	6	0	1.423327	1.858692	-1.381534
4	6	0	0.874552	0.984673	-0.382152
5	6	0	-0.050247	1.625679	0.410960
6	7	0	0.405733	-1.371820	0.068507
7	7	0	-0.818379	1.010763	1.397225
8	6	0	-1.329993	-0.260027	1.269028
9	6	0	-1.024262	-1.067147	0.053845
10	7	0	-2.002096	-0.580760	2.332916
11	6	0	-1.246348	1.438014	2.634172
12	7	0	-1.953173	0.492311	3.192576
13	6	0	1.242308	4.312123	-2.149685
14	6	0	-0.937468	2.752337	3.241667
15	6	0	1.244062	-0.430742	-0.156030

16	6	0	3.637812	0.121567	0.347444
17	6	0	2.685965	-0.786246	-0.114683
18	6	0	3.102256	-2.066189	-0.484064
19	6	0	4.434295	-2.432196	-0.402608
20	6	0	5.360620	-1.509946	0.065804
21	6	0	4.974052	-0.234631	0.446994
22	17	0	7.037838	-1.962928	0.173784
23	6	0	2.400552	1.443606	-2.438025
24	6	0	-1.808002	-2.370984	0.013450
25	6	0	-3.309029	-2.267005	-0.135541
26	8	0	-4.059185	-3.137492	0.237001
27	8	0	-3.671022	-1.146073	-0.760866
28	6	0	-5.078360	-0.831049	-1.063484
29	6	0	-5.648202	-1.871787	-2.016343
30	6	0	-4.979817	0.524290	-1.746809
31	6	0	-5.878805	-0.721382	0.226207
32	1	0	-1.271873	-0.456908	-0.823414
33	1	0	2.276100	4.632931	-2.002256
34	1	0	0.595594	5.158527	-1.921384
35	1	0	1.118759	4.078332	-3.209433
36	1	0	-1.455269	3.568279	2.733767
37	1	0	0.132723	2.963664	3.211771
38	1	0	-1.264669	2.733339	4.278983
39	1	0	3.334158	1.115209	0.651995
40	1	0	2.367390	-2.776025	-0.839338
41	1	0	4.754638	-3.421610	-0.699589
42	1	0	5.706461	0.468972	0.818373
43	1	0	2.272447	0.395817	-2.708080
44	1	0	2.268446	2.041271	-3.340136
45	1	0	3.434743	1.570320	-2.109630
46	1	0	-1.446854	-2.953354	-0.838723
47	1	0	-1.609590	-2.961901	0.905947
48	1	0	-5.022254	-1.957998	-2.906172
49	1	0	-5.724809	-2.846631	-1.540682
50	1	0	-6.644596	-1.559068	-2.332898
51	1	0	-4.535332	1.262056	-1.077709
52	1	0	-5.975728	0.868919	-2.027138
53	1	0	-4.370466	0.459448	-2.649215
54	1	0	-5.401523	-0.021029	0.913135
55	1	0	-6.875917	-0.343303	-0.005344
56	1	0	-5.978975	-1.685643	0.718370

Conformer # 5

Energy: -2119.270848 Hartree

Center Number	Atomic Number	Atomic Type	Coordinates (Angstroms) X	Y	Z
1	16	0	-1.914903	-3.678151	-0.071035
2	6	0	-3.136843	-2.871510	-0.990989
3	6	0	-2.851994	-1.545308	-1.161783
4	6	0	-1.623954	-1.159616	-0.522357
5	6	0	-1.009991	-2.223625	0.096642
6	7	0	0.181014	0.484894	-0.674600
7	7	0	0.244535	-2.183657	0.702010
8	6	0	1.302444	-1.455562	0.205661
9	6	0	1.118187	-0.585652	-0.992824
10	7	0	2.359282	-1.654549	0.933130
11	6	0	0.755011	-2.840532	1.798782
12	7	0	2.015175	-2.527200	1.937922
13	6	0	-4.316007	-3.640916	-1.487727
14	6	0	-0.006874	-3.741306	2.693695
15	6	0	-1.051921	0.205116	-0.482885
16	6	0	-1.689366	2.615127	-0.634812
17	6	0	-1.952071	1.337064	-0.139750
18	6	0	-3.029250	1.161582	0.727384
19	6	0	-3.828200	2.232584	1.098409
20	6	0	-3.551019	3.488679	0.582754
21	6	0	-2.486178	3.691048	-0.285081
22	17	0	-4.556177	4.838011	1.030858
23	6	0	-3.715386	-0.634043	-1.979253
24	6	0	2.437168	-0.050373	-1.538261
25	6	0	3.296740	0.772577	-0.597247
26	8	0	2.887290	1.525455	0.250470
27	8	0	4.584849	0.579488	-0.904147
28	6	0	5.670048	1.300841	-0.223013
29	6	0	5.539887	2.797279	-0.471582
30	6	0	6.915356	0.753516	-0.904630
31	6	0	5.679164	0.956373	1.259535
32	1	0	0.685395	-1.222652	-1.779360
33	1	0	-4.426093	-3.528042	-2.568617
34	1	0	-5.241720	-3.291704	-1.024719
35	1	0	-4.218878	-4.705116	-1.276091
36	1	0	-0.936853	-3.278675	3.028040
37	1	0	-0.257026	-4.685907	2.207054

38	1	0	0.611199	-3.958997	3.562129
39	1	0	-0.848033	2.757723	-1.299598
40	1	0	-3.240605	0.181183	1.135468
41	1	0	-4.655021	2.093902	1.781478
42	1	0	-2.285937	4.678134	-0.679117
43	1	0	-4.476215	-0.138783	-1.371581
44	1	0	-4.232666	-1.192435	-2.759745
45	1	0	-3.125595	0.146097	-2.459520
46	1	0	3.032378	-0.879140	-1.918108
47	1	0	2.208538	0.593302	-2.392160
48	1	0	6.423999	3.303742	-0.080566
49	1	0	5.479334	3.001981	-1.541970
50	1	0	4.659785	3.207697	0.017879
51	1	0	6.891101	0.958191	-1.975829
52	1	0	6.991543	-0.324696	-0.758099
53	1	0	7.804946	1.223069	-0.483286
54	1	0	5.706700	-0.125323	1.397887
55	1	0	4.803342	1.353686	1.766588
56	1	0	6.572977	1.380989	1.719979

Conformer # 6

Energy: -2119.270031 Hartree

Center Number	Atomic Number	Atomic Type	Coordinates (Angstroms) X	Y	Z
1	16	0	-0.041559	3.196122	1.783259
2	6	0	0.468883	1.866228	2.761912
3	6	0	0.737848	0.756342	2.009443
4	6	0	0.544363	0.984681	0.603028
5	6	0	0.114227	2.264258	0.345254
6	7	0	0.001943	-0.214579	-1.478013
7	7	0	-0.274853	2.744542	-0.903860
8	6	0	-0.926107	1.987938	-1.850652
9	6	0	-1.236557	0.538944	-1.674732
10	7	0	-1.150821	2.705221	-2.912419
11	6	0	-0.131008	3.985909	-1.482748
12	7	0	-0.656220	3.964891	-2.678010
13	6	0	0.571958	2.034929	4.241740
14	6	0	0.531716	5.154541	-0.860024
15	6	0	0.765353	-0.009498	-0.472919
16	6	0	3.202423	-0.272968	0.067492

17	6	0	2.016803	-0.813668	-0.426984
18	6	0	2.034488	-2.106534	-0.951470
19	6	0	3.202246	-2.848809	-0.974742
20	6	0	4.370715	-2.284176	-0.480988
21	6	0	4.384046	-0.998395	0.036138
22	17	0	5.845834	-3.208790	-0.511416
23	6	0	1.133802	-0.551940	2.622447
24	6	0	-2.323699	0.255412	-0.632013
25	6	0	-2.900572	-1.138638	-0.772577
26	8	0	-2.861011	-1.792276	-1.787107
27	8	0	-3.485151	-1.503043	0.368116
28	6	0	-4.222726	-2.772374	0.506062
29	6	0	-4.714201	-2.717522	1.944294
30	6	0	-3.277589	-3.948309	0.306688
31	6	0	-5.397832	-2.798864	-0.460582
32	1	0	-1.638524	0.217258	-2.635353
33	1	0	1.610319	1.977203	4.576349
34	1	0	0.170498	2.994621	4.565125
35	1	0	0.015336	1.252080	4.761423
36	1	0	0.648951	5.922737	-1.621419
37	1	0	1.515253	4.894088	-0.465730
38	1	0	-0.059149	5.571424	-0.042193
39	1	0	3.213242	0.734033	0.465504
40	1	0	1.117743	-2.525685	-1.344064
41	1	0	3.209861	-3.854911	-1.371556
42	1	0	5.304755	-0.569640	0.407513
43	1	0	0.769412	-1.393840	2.034894
44	1	0	2.218507	-0.655220	2.699232
45	1	0	0.724493	-0.643265	3.628673
46	1	0	-3.154967	0.949136	-0.790990
47	1	0	-1.982686	0.405332	0.389883
48	1	0	-3.874214	-2.656862	2.637456
49	1	0	-5.286703	-3.616877	2.173379
50	1	0	-5.356884	-1.850038	2.099447
51	1	0	-2.415350	-3.860657	0.969649
52	1	0	-2.929110	-4.011767	-0.721369
53	1	0	-3.801277	-4.873135	0.554049
54	1	0	-6.024201	-1.915899	-0.323550
55	1	0	-6.007197	-3.680510	-0.255760
56	1	0	-5.064864	-2.841008	-1.495005

CompareVOA results (cc-pVTZ / B3PW91):

Optimal Scaling Factor = 0.980

S_{fg} (IR) = 83.9

S_{fg} (VCD S-config) = 85.6

S_{fg} (VCD R-config) = 2.5

ESI (Enantiomeric Similarity Index) = 83.1

Confidence Level = 100

References

1 G. M. Sheldrick, *Acta Crystallogr. Sect. C Struct. Chem.*, 2015, **71**, 3.

2 A. L. Spek, *J. Appl. Crystallogr.*, 2003, **36**, 7.

3 O. V. Dolomanov, L. J. Bourhis, R. J. Gildea, J. A. K. Howard and H. Puschmann, *J. Appl. Crystallogr.*, 2009, **42**, 339.

4 L. J. Farrugia, *J. Appl. Crystallogr.*, 2012, **45**, 849.

5 C. F. Macrae, P. R. Edgington, P. McCabe, E. Pidcock, G. P. Shields, R. Taylor, M. Towler and J. Van De Streek, *J. Appl. Crystallogr.*, 2006, **39**, 453.

6. He, Y.; Wang, B.; Dukor, R. K.; Nafie, L. A. *Applied Spectroscopy* **2011**, *65*, 699.

7. Bogaerts, J.; Aerts, R.; Vermeyen, T.; Johannessen, C.; Herrebout, W.; Batista, J.M. *Pharmaceuticals* **2021**, *14*(9), 877.

8. O'Connor , T.J.; Mai, B.K.; Nafie, J.; Liu, P.; Toste, F.D. *J. Am. Chem. Soc.* **2021**, *143*, 13759.

9. Stephens, P. J.; Devlin, F. J.; Cheeseman, J. R.; Frisch, M. J. *J. Phys. Chem. A* **1997,** *101,* 6322.

10. Polavarapu, P. L.; Covington, C. L. *Chirality* **2014,** *26*, 539-552.

11. Debie, E.; DeGuessem E.; Dukor, R. K.; Herrebout, W.; Nafie, L. A.; Bultinck, P. *ChemPhysChem* **2011,** *12,* 1542.
